# Curcumin Protected CoCl_2_ ‐Induced Apoptosis and Ferroptosis in Human Umbilical Vein Endothelial Cells by Regulating the Expression of HSPA6

**DOI:** 10.1002/fsn3.71343

**Published:** 2026-01-05

**Authors:** Ruihai Ye, Hang Lv, Zhe Ren, Xiaohong Yang, Xin Lv, Xue Xu

**Affiliations:** ^1^ Department of Respiratory and Critical Care Medicine Zhejiang Provincial Hospital of Chinese Medicine Hangzhou Zhejiang Province China; ^2^ Clinical Laboratory Zhejiang Provincial Hospital of Chinese Medicine Hangzhou Zhejiang Province China; ^3^ Department of Thoracic Surgery Zhejiang Provincial Hospital of Chinese Medicine Hangzhou Zhejiang Province China; ^4^ Department of Dermatology Zhejiang Provincial Hospital of Chinese Medicine Hangzhou Zhejiang Province China; ^5^ Physiatry Department Zhejiang Provincial Hospital of Chinese Medicine Hangzhou Zhejiang Province China

**Keywords:** curcumin, ferroptosis, hypoxia, vascular endothelial cell

## Abstract

Hypoxia‐induced vascular endothelial cell (EC) injury is the main pathologic mechanism for the development of vascular diseases, such as venous thrombosis, heart disease, and cerebral obstruction. Curcumin, the main active component in the rhizome of *Curcuma longa* , has anti‐inflammatory and antioxidant properties. The aim of the study was to elucidate the underlying mechanisms of curcumin's protective effects on endothelial cells. Cobalt chloride (CoCl_2_) was used to induce hypoxia in human umbilical vein endothelial cells (HUVECs) in vitro, followed by treatment with curcumin. We found that curcumin can enhance cell proliferation, promote cell cycle progression, decrease MMP1 and MMP13 expression, and increase TIMP‐1 expression in CoCl_2_‐induced HUVECs. Meanwhile, curcumin inhibits CoCl_2_‐induced apoptosis, ferroptosis, and mitochondrial damage in HUVECs. Further studies revealed that curcumin exerted endothelial cell protective effects by down‐regulating HSPA6 expression. Curcumin exerts a vascular endothelial protective effect on hypoxia‐induced apoptosis and mitochondrial damage in HUVECs. Thus, curcumin is very effective for the clinical treatment of hypoxia‐induced vascular diseases, such as cardiovascular diseases, venous thrombosis, and so on.

## Graphical abstract

Curcumin reduces the expression of MMP1 and MMP13 while increasing TIMP‐1 levels by downregulating HSPA6 in CoCl_2_‐induced HUVECs. This mechanism attenuates apoptosis, ferroptosis, and mitochondrial damage in HUVECs. These findings suggest that curcumin holds promise for the clinical treatment of hypoxia‐induced vascular diseases.

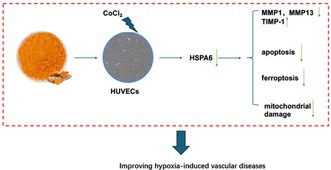

## 1 Introduction

Hypoxia affects cells and tissues during embryonic development, wound healing, organ function (Burtscher et al. 2021; Mallat et al. 2022). Vascular endothelial cells are the primary receptors of organ hypoxia (Zhou et al. 2020). Hypoxia plays an important role in protective and pathological vascular adaptation. Hypoxia leads to vascular endothelial cell damage and dysfunction, thereby induces blood flow disturbances (Giannarelli et al. 2014; Mao et al. 2013; Zhao et al. 2015). It is generally believed that hypoxia‐induced vascular endothelial cell injury is the main pathological mechanism for the development and progression of many diseases, such as cardiovascular and cerebrovascular diseases (Conti et al. 2017; Huang et al. 2022; Nitzsche et al. 2021), diabetes mellitus (Li, Yuan, et al. 2023), as well as venous thrombosis (Chen et al. 2016). Consequently, preventing hypoxia‐induced vascular endothelial damage and elucidating its molecular mechanisms are vital for improving the treatment of cardiovascular and cerebrovascular diseases, venous thrombosis, and other vascular conditions.

Under hypoxic conditions, the massive production of reactive oxygen species triggers extensive apoptosis and death of vascular endothelial cells, which lead to vascular endothelial injury (Hu et al. 2018; Kondoh et al. 2013; Zhou et al. 2020). Recent studies have shown that ferroptosis is closely related to this process (Li et al. 2021). Ferroptosis is an iron‐dependent regulatory mechanism of cell death, characterized by increased intracellular iron and decreased antioxidant capacity, leading to lipid peroxides (LPO) accumulation (Zheng et al. 2023). Relevant studies have indicated that hypoxia regulates ferroptosis (Gao et al. 2023). It has been found that hypoxia can induce ferroptosis in cerebrovascular endothelial cells and cause blood–brain barrier disruption (Liu et al. 2023). Chen et al. discovered that after the treatment of hypoxia‐induced arterial endothelial cells with Fer‐1 (an inhibitor of ferroptosis), the mitochondrial damage and arterial endothelial cell injury are obviously relieved (Chen et al. 2024). Previous studies have shown that erastin, an activator of ferroptosis, could exacerbate the ferroptosis process in vascular endothelial cells, worsen endothelial cell injury and result in myocardial ischemia (Fang et al. 2023). Therefore, blocking the ferroptosis process is a novel approach to safeguard vascular endothelial cells from hypoxia‐induced injury.

Curcumin is a naturally occurring polyphenolic compound found in the rhizome of the traditional Chinese medicine *Curcuma longa* (Kotha and Luthria 2019). It possesses a variety of pharmacological activities, including antioxidative stress (Cao et al. 2020), anti‐inflammatory (Peng et al. 2021), and anti‐tumor effects (Ming et al. 2022). It has been reported to have a protective effect on the vascular endothelium. Zhao et al. found that curcumin can reduce endothelial lipotoxicity induced by highly oxidized low‐density lipoprotein (ox‐LDL) by inducing autophagy in human umbilical vein endothelial cells (HUVECs) and protecting endothelial cells from damage (Zhao et al. 2021). Curcumin improves HUVEC function by restoring αvβ3 integrin and decreasing endothelin‐1 expression, as well as inhibiting H_2_O_2_‐induced cellular pyroptosis (Yuan et al. 2022). In conclusion, curcumin may have the potential for the treatment of diseases related to endothelial cell dysfunction. Curcumin also has a regulatory role in ferroptosis. Curcumin induces ferroptosis in cancer cells while serving as an inhibitor of ferroptosis in tissue injury (Foroutan et al. 2024). For example, curcumin attenuates kidney injury associated with rhabdomyolysis by reducing ferroptosis‐mediated cell death (Guerrero‐Hue et al. 2019). Accordingly, the mechanism of ferroptosis regulation by curcumin needs to be further elucidated. The aim of the present study is to investigate whether curcumin could mitigate hyperoxia‐induced vascular endothelial cell injury by modulating the ferroptosis pathway.

## 2 Materials and Methods

### 2.1 Cell Culture

Immortalized human umbilical vein endothelial cells (HUVECs) were obtained from the Chinese Academy of Sciences Cell Bank (China). HUVECs were cultured in DMEM‐F12 (Gibco, USA) supplemented with 10% heat‐inactivated fetal bovine serum, 100 U/mL penicillin, 100 U/mL streptomycin (Thermo Fisher Scientific, USA), 30 μg/mL endothelial cell growth supplement (Gibco; Thermo Fisher Scientific, USA), and 5 U/mL heparin (Thermo Fisher Scientific, USA) and 5 U/mL heparin (Thermo Fisher Scientific, USA) at 37°C with a concentration of 5% CO_2_. All were STR‐validated after purchase to ensure the authenticity of the cell lines, and experiments were performed within 10 generations to maintain the integrity of the cells. In addition, all cells were not contaminated with mycoplasma.

### 2.2 Cell Transfection

The plasmid vector overexpressing HSPA6 and the negative control vector were synthesized by GeneChem (China). They were transfected into HUVECs using Lipofectamine 3000 (Thermo Fisher Scientific, USA) according to the manufacturer's instructions.

### 2.3 Drug Treatment

The cells used in this study were at passage 3 and 4. To simulate the hypoxic/ischemic environment of endothelial cells, HUVECs were pre‐incubated with 100 μM CoCl_2_ for 12 h until 90% confluence. After incubation, the cells were washed twice with phosphate buffer (pH 7.4) and then exposed to different concentrations (10, 20, 50, and 100 μM) of curcumin (Cur, Sigma Aldrich, USA) for 12 h to explore the function of curcumin.

In the rescue experiment, all groups of HUVECs were treated with CoCl_2_. For the group overexpressing HSPA6, the plasmid vector was transfected for 48 h before treatment with CoCl_2_, curcumin, or Ferrostatin‐1 (Fer‐1). For the Fer‐1‐treated group, HUVECs were pre‐incubated with CoCl_2_ for 12 h and then treated with 1 μM Fer‐1 (MedChemExpress, USA) for 12 h.

### 2.4 Cell Counting Kit 8 (CCK‐8)

Cell counting kit‐8 (CCK‐8, Beyotime, China) was used to analyze cell viability. When the HUVECs confluence reached 85%, the cells of each group were seeded into a 96‐well microtiter plate (2000 cells per well) and cultured in 100 μL of culture medium. After 1–5 days of culture, the cells were incubated with 10 μL of CCK‐8 reagent/well for 2 h. The absorbance was then analyzed at 450 nm using a spectrophotometer (Multiscan MK3, Thermo Fisher Scientific, Waltham, MA, USA).

### 2.5 Quantitative Reverse Transcription PCR (RT‐qPCR)

Following the manufacturer's instructions, RNA was extracted from HUVECs using Trizol reagent (TaKaRa Biotech, Japan), and MultiScribe reverse transcriptase (Applied Biosystems, USA) was used to convert 4 μg of total RNA to cDNA. cDNA was amplified using the Fast SYBR Green Master Mix (Applied Biosystems, USA). The LightCycler 96 (Roche, USA) was used to perform qPCR. The primer sequences used in this study were shown in Table 1. The mRNA expression was quantitated using the 2‐ (△C_T_ sample − △C_T_ control) method.

**TABLE 1:** Table list of all primers used RT‐qPCR.

Species	Primer	Sequence (5′‐3′)	Amplification fragment size (bp)
*Homo sapiens* (human)	GAPDH‐F	TGACTTCAACAGCGACACCCA	121
	GAPDH‐R	CACCCTGTTGCTGTAGCCAAA	
	MMP1‐F	GGCTGAAAGTGACTGGGAAAC	163
	MMP1‐R	GGCAAATCTGGCGTGTAA	
	MMP13‐F	CGACTTCTACCCATTTGA	112
	MMP13‐R	ACTACTTGTCCAGGTTTCA	
	TIMP‐1‐F	TTCCACAGGTCCCACAAC	165
	TIMP‐1‐R	GCATTCCTCACAGCCAAC	
	COL1A1‐F	AAGGTGTTGTGCGATGACG	116
	COL1A1‐R	TGGTCGGTGGGTGACTCTG	
	COL3A1‐F	AGCTTCCCAGAACATCACA	130
	COL3A1‐R	AGCTTCCCAGAACATCACA	
	HSPA6_F	GGGACAAATGTGAGAAAGTGC	149
	HSPA6_R	GTAGGTGGTGAAAGTCTGGGT	
	POSTN‐F	GAGACAAAGTGGCTTCCG	134
	POSTN‐R	GAGACAAAGTGGCTTCCG	
	THBS2_F	CCCGACATCTGCTTCTCC	136
	THBS2_R	TTTCCGTCTTGGCTGCTT	
	VCAN_F	TTTCAGCCAACTACATCA	147
	VCAN_R	CACATCTTCTACTTCACCC	

### 2.6 Flow Cytometry Assay

When the HUVECs confluence reaches 85%, the apoptotic level of cells in each group is tested by flow cytometry using the Annexin‐V‐FITC/PE Apoptosis Detection Kit (Beyotime, China) according to the manufacturer's guidelines, and the apoptotic rate was calculated as the sum of the proportions of early and later apoptotic cells in the total number of cells.

The cell cycle was analyzed using a cell cycle kit (MUSE, Millipore, USA). HUVECs stained with Propidium iodide (PI), and PI‐positive cells were analyzed according to the manufacturer's protocol.

### 2.7 Western Blot Analysis (WB)

RIPA lysis buffer (Beyotime, China) was used to extract the total protein from HUVECs. Subsequently, equivalent volumes of protein extracts were loaded onto a PVDF membrane (0.45 μm; Millipore, USA) using sodium dodecyl sulfate‐polyacrylamide gel electrophoresis (SDS‐PAGE). The membranes were then blocked for an hour at room temperature with 5% skim milk, and incubated with primary antibodies overnight at 4°C. After three washes with TBST, the membranes were incubated for an hour at room temperature with HRP‐labeled secondary antibodies (1:3000). The membrane was examined using the ECL protein blotting assay (Beyotime, China) following an additional wash. The primary antibodies were shown in Table 2. The original Western blot images are available in the Data S1.

**TABLE 2:** Antibody information.

Gene name	Catalog number	Manufacturer	Dilution ratios
FTH1	ab183781	abcam	1:2000
GPX4	ab125066	abcam	1:2000
ACSL4	ab155282	abcam	1:2000
HSPA6	ab212044	abcam	1:2000
GAPDH	AF7021	Affinity	1:2000
Goat anti‐rabbit	AF7021	Affinity	1:3000

### 2.8 Enzyme‐Linked Immunosorbent Assay (ELISA)

Intracellular iron content was measured using an iron assay kit (ab83366, Abcam, UK) according to the manufacturer's instructions. The optical density (OD) at 450 nm was assessed using a microplate reader (Thermo Fisher, Waltham, MA, USA).

### 2.9 Detection of Mitochondrial Membrane Potential

The mitochondrial membrane potential was detected using JC‐10 kits (CA1310, Solarbio Science, China). A total of 10 μg/mL JC‐10 was added to medium solution, the cells were incubated at 37°C for 30 min and washed with PBS. Cells were then observed under a fluorescence microscope (Zeiss, Germany), and images of green and red fluorescence intensities in each cell were analyzed. The intensity ratio of red fluorescence to green fluorescence is the mitochondrial membrane potential.

### 2.10 Transcriptome Sequencing

Total RNA from HUVECs was extracted using Trizol reagent (Invitrogen, USA) and subsequently subjected to RNA‐seq. RNA was quantified using the Qubit RNA HS Assay Kit (Thermo Fisher Scientific, USA) and diluted to 100 ng/μL. The quality of total RNA was confirmed using a Fragment Analyzer (Advanced Analytical Technologies, Germany). Samples with RNA integrity number (RIN) ≥ 6 were selected for RNA library preparation according to the ISO/IEC 17025‐approved protocol (TruSeq RNA Library Preparation Kit v2, Illumina, USA). mRNA with polyA tails was enriched from total RNA using Oligo (dT) magnetic beads, fragmented in Fragmentation Buffer containing divalent cations, and then subjected to cDNA synthesis, adapter ligation, and PCR amplification. Finally, the RNA‐Seq data sequence analysis of the 150 bp paired‐end reads was performed using the Illumina NovaSeq 6000 platform. Image analysis, base calling, and quality checks were performed using the Illumina data analysis pipeline RTA v1.18.64, and the Bcl2fastq v1.8.4. RNAseq reads were provided in compressed Sanger FASTQ format.

Differentially expressed genes (DEGs) were analyzed using the DESeq2 (v1.20.0) or edgeR (v3.22.5). The P value was adjusted using the Benjamini & Hochberg method. A criteria of *P*_adj_ ≤ 0.05 &|log2 (foldchange)| ≥ 1 was set for the judgment of significant differential expression.

## 3 Statistical Analysis

Images were created using GraphPad Prism 8 (GraphPad Software Inc., USA), and the data were statistically analyzed using SPSS 26.0 (IBM Corporation, USA). A one‐way ANOVA with post hoc Bonferroni corrections for multiple comparisons was used to identify statistically significant differences. Data are presented as mean ± standard deviation (SD). The criterion for statistical significance was set at *p* < 0.05.

## 4 Results

### 4.1 Curcumin Enhances Proliferation and Inhibits Apoptosis in CoCl_2_ ‐Induced HUVECs

**FIGURE 1:**
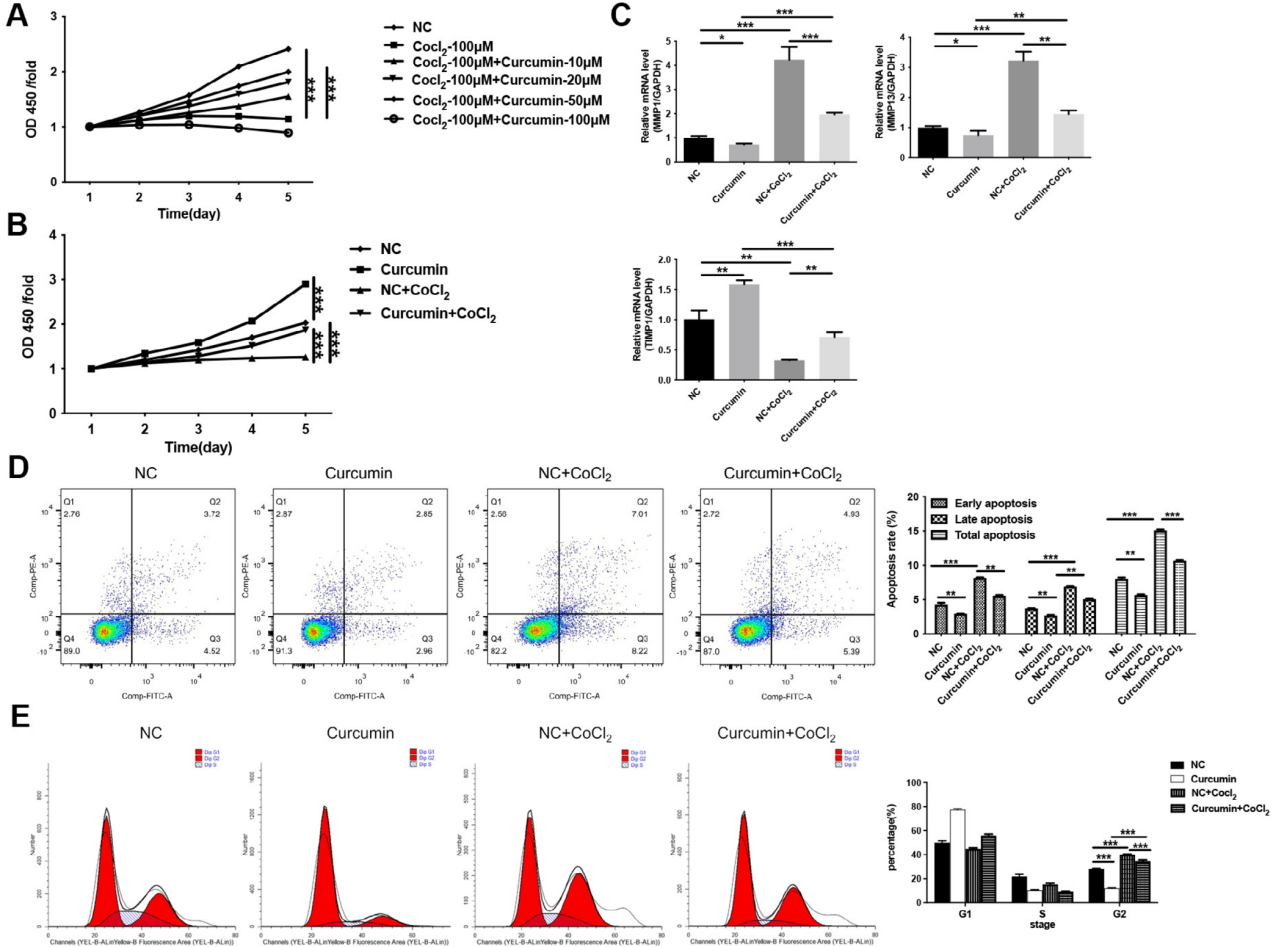
Curcumin enhanced proliferation and inhibited apoptosis in CoCl_2_‐induced HUVECs. Following pre‐incubation with 100 μM CoCl_2_ for 12 h, the HUVECs were treated with curcumin. (A) CCK8 was used to assess cell viability after treatment of HUVECs with different concentrations of curcumin. (B) CCK8 was used to assess cell viability after treatment of HUVECs with CoCl_2_ or 20 μM curcumin. (C) The mRNA expression of MMP1, MMP13 and TIMP‐1 was measured by RT‐qPCR. (D) Apoptosis was analyzed by flow cytometry. (E) Cycle progression was analyzed by flow cytometry. *p < 0.05, **p < 0.01, ***p < 0.001.

To investigate the therapeutic effect of curcumin on vascular endothelial cell injury, human umbilical vein endothelial cells (HUVECs) were treated with CoCl_2_ to simulate the vascular hypoxic/ischemic environment. Subsequently, HUVECs were treated with different concentrations of curcumin. We found that CoCl_2_ significantly reduced HUVEC viability (*p* < 0.001), whereas curcumin reversed this reduction in cell viability within the 10–50 μM concentration range (*p* < 0.001; Figure 1A). Therefore, a medium concentration (20 μM) of curcumin was selected for subsequent studies. As shown in Figure 1B, 20 μM curcumin increases cell viability compared to the negative control (NC) group and significantly enhances CoCl_2_‐induced cell viability (*p* < 0.001). Dysregulation of matrix metalloproteinases (MMPs) and their inhibitors, the markers of vascular endothelial injury, was also evaluated. RT‐qPCR results show that CoCl_2_ significantly upregulates the expression of MMP1 and MMP13 (*p* < 0.001) and decreases the expression of TIMP‐1 (*p* < 0.01). In contrast, curcumin treatment decreases the expression of MMP1 and MMP13 and increases the expression of TIMP‐1 (*p* < 0.01; Figure 1C). This indicates that curcumin regulates MMP's activity and extracellular matrix degradation in HUVECs. Furthermore, flow cytometry was used to assess the effect of curcumin on apoptosis and cell cycle progression in HUVECs. The results showed that CoCl_2_ dramatically promoted apoptosis in HUVECs (*p* < 0.001), and curcumin reversed this effect (*p* < 0.001; Figure 1D). CoCl_2_ also induces cell cycle arrest in the G2 phase, whereas treatment with curcumin promotes cell cycle progression (Figure 1E). In conclusion, these results suggest that curcumin improves hypoxia‐induced HUVEC injury by increasing cell proliferation and cell cycle progression, as well as decreasing apoptosis.

### 4.2 Inhibition of Ferroptosis and Amelioration of Mitochondrial Collapse by Curcumin in CoCl_2_ ‐Induced HUVECs

**FIGURE 2:**
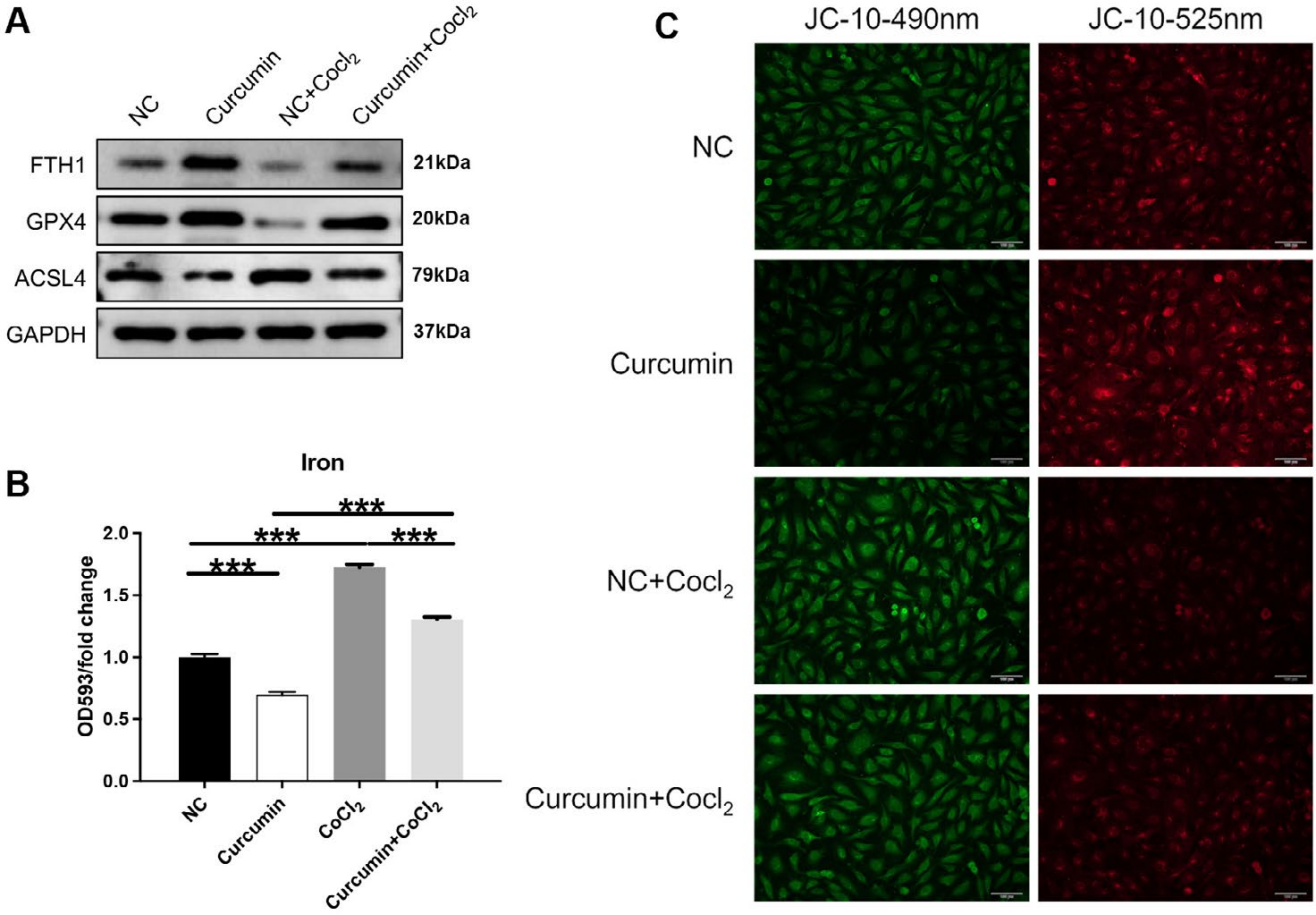
Inhibition of ferroptosis and amelioration of mitochondrial collapse by curcumin in CoCl_2_‐induced HUVECs. Following pre‐incubation with 100 μM CoCl_2_ for 12 h, the HUVECs were treated with 20 μM curcumin for 12 h. (A) The protein expression of FTH1, GPX4, and ACSL4 was determined by western blotting. (B) Iron levels in cells were determined by ELISA. (C) Mitochondrial membrane potential was assessed by JC‐10 staining (Scale bar: 100 μm). ***p < 0.001.

Ferroptosis, characterized by iron accumulation and lipid peroxidation, represents an oxidative stress‐dependent regulation of cell death pathways. Under hypoxia, the process of cellular ferroptosis may be stimulated. In this study, we found that CoCl_2_ induced an increase in the expression of the ferroptosis protein ACSL4 and a decrease in the expression of the anti‐ferroptosis proteins FTH1 and GPX4 in HUVECs (Figure 2A). However, curcumin treatment decreases the expression of ACSL4 and increases the expression of FTH1 and GPX4 in CoCl_2_‐induced HUVECs (*p* < 0.001; Figure 2B). Additionally, curcumin diminishes iron levels in cells, whereas CoCl_2_ increases iron levels compared to the NC group. Simultaneous treatment with curcumin reversed the CoCl_2_‐induced increase in iron levels (*p* < 0.001; Figure 2B).

Changes in mitochondrial membrane potential during cell death were analyzed using JC‐10 staining. The results showed that CoCl_2_ treatment can increase the intensity of green fluorescence and decrease the intensity of red fluorescence in the cells, indicating increased cellular damage. In contrast, 20 μM curcumin reversed the CoCl_2_‐induced changes in JC‐10 fluorescence (Figure 2C). In conclusion, these results reveal that curcumin reduces CoCl_2_‐induced ferroptosis and mitochondrial damage in HUVECs.

### 4.3 Identification of Downstream Differentially Expressed Genes Regulated by Curcumin Based on Transcriptomic Data

**FIGURE 3:**
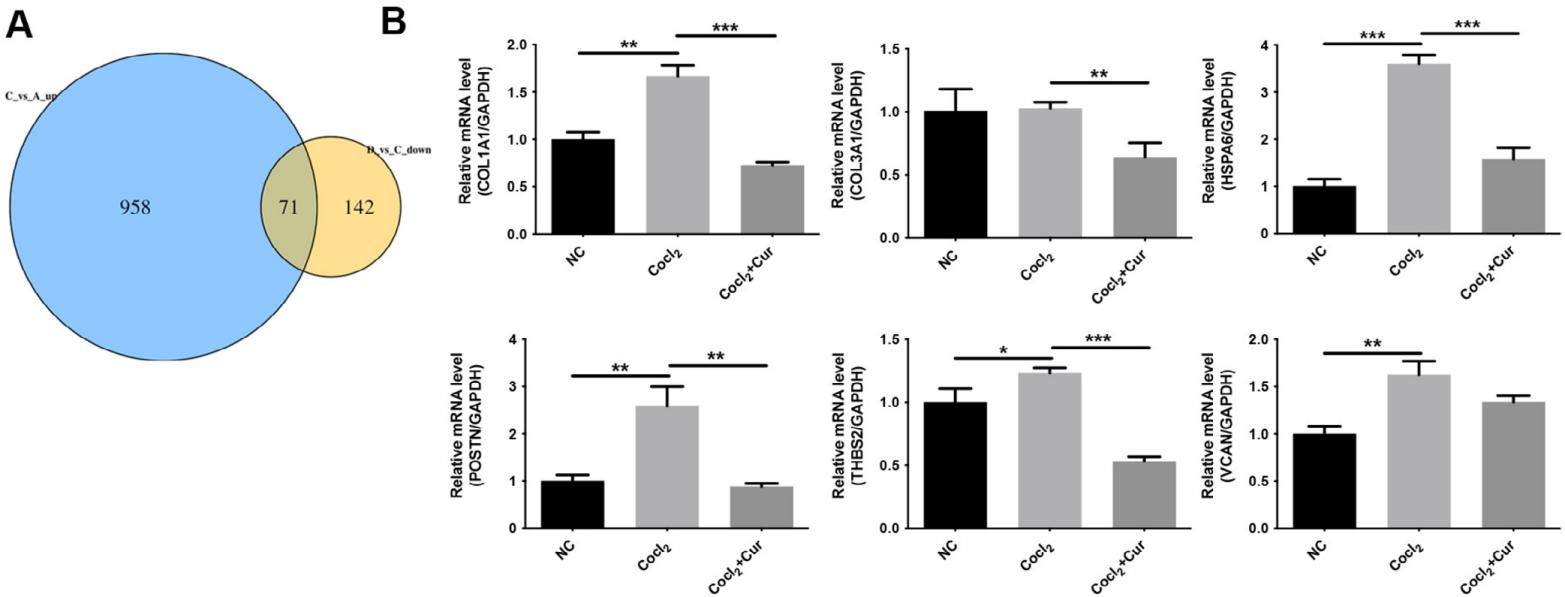
Identification of downstream differentially expressed genes regulated by curcumin based on transcriptomic data. Following pre‐incubation with 100 μM CoCl_2_ for 12 h, the HUVECs were treated with 20 μM curcumin for 12 h. (A) Venn diagram of differentially expressed genes. A represents the group of untreated HUVECs; C represents HUVECs treated with CoCl_2_; and D represents HUVECs treated with CoCl_2_ and curcumin. (B) The mRNA expression of COL1A1, COL3A1, THBS2, HSPA6, VCAN, and POSTN was measured by RT‐qPCR. *p < 0.05, **p < 0.01, ***p < 0.001.

To investigate the specific mechanism by which curcumin alleviates endothelial cell injury, a transcriptome sequencing method was used. Three treatment groups, namely, the NC group, the CoCl_2_ group, and the CoCl_2_ + curcumin group, were selected for transcriptome sequencing. The results showed that a total of 1029 genes were significantly upregulated by CoCl_2_ compared to the NC group. A total of 213 genes were significantly downregulated by curcumin compared to the CoCl_2_ group. After identifying genes related to curcumin efficacy, we found 71 genes that were both upregulated by CoCl_2_ and suppressed by curcumin (Figure 3A). Among these genes, COL1A1, COL3A1, THBS2, HSPA6, VCAN, and POSTN are the six genes with the most significant changes. These six genes were subsequently validated in HUVECs cells using RT‐qPCR. We found that HSPA6 expression was significantly elevated in the CoCl_2_ group compared to the NC group (*p* < 0.001), and curcumin dramatically reduced HSPA6 expression induced by CoCl_2_ (*p* < 0.001; Figure 3B). Therefore, HSPA6 was selected for further analysis.

### 4.4 Overexpression of HSPA6 Reversed the Function of Curcumin in CoCl_2_ ‐Induced HUVECs

**FIGURE 4:**
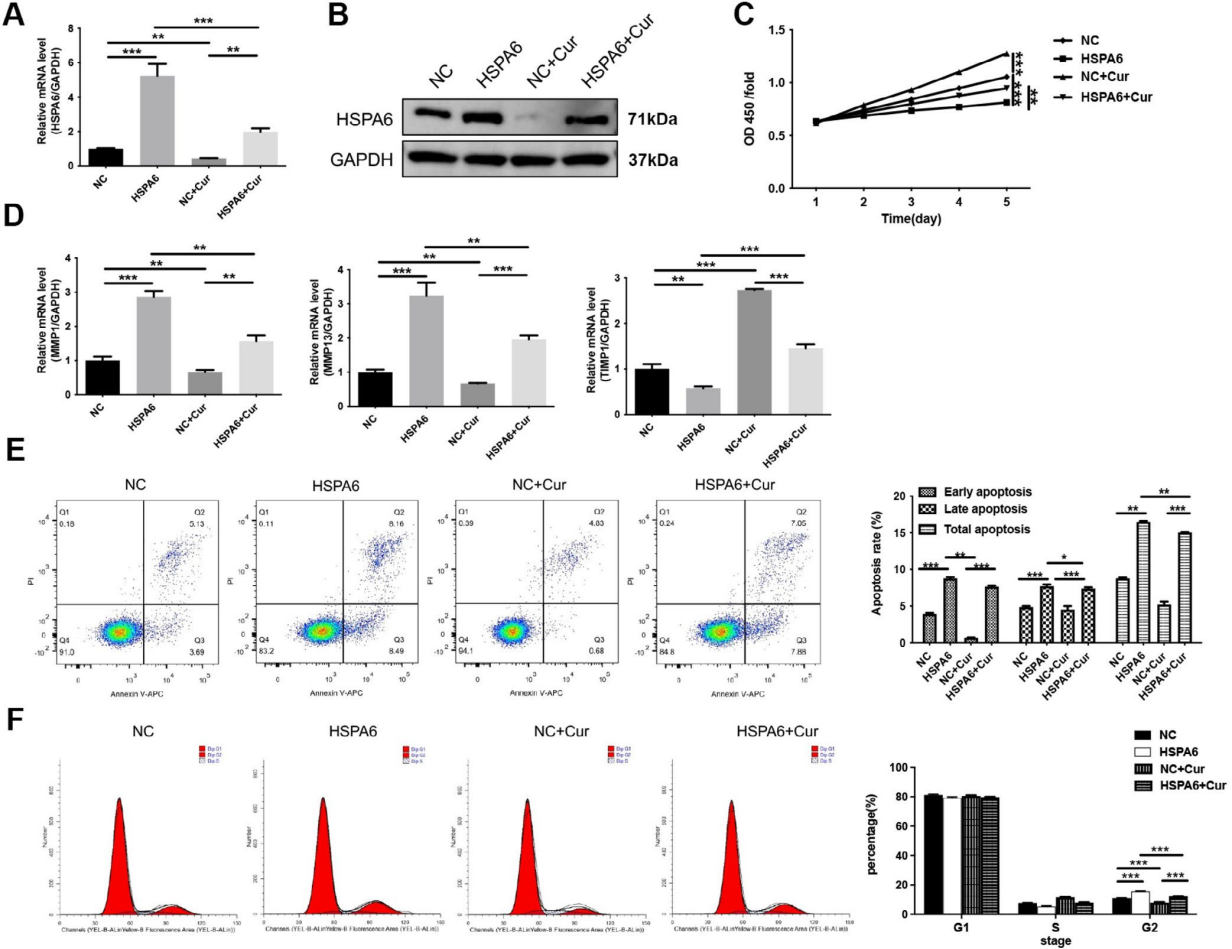
Curcumin enhanced proliferation in CoCl_2_‐induced HUVECs by HSPA6. The HUVECs were transfected with HSPA6 vector and were subsequently pre‐incubated with 100 μM CoCl_2_ for 12 h, followed by 20 μM curcumin for 12 h. (A) The mRNA expression of HSPA6 was measured by RT‐qPCR. (B) The protein expression of HSPA6 was determined by western blotting. (C) CCK8 was used to assess cell viability. (D) The mRNA expression of MMP1, MMP13 and TIMP‐1 was measured by RT‐qPCR. (E) Apoptosis was analyzed by flow cytometry. (F) Cycle progression was analyzed by flow cytometry. *p < 0.05, **p < 0.01, ***p < 0.001.

To investigate whether curcumin exerts endothelial cell protection by regulating HSPA6, plasmids overexpressing HSPA6 were transfected into HUVEC cells, and the cells were subsequently treated with CoCl_2_. As shown in Figure 4A,B, HSPA6 expression is significantly increased in cells transfected with the plasmid overexpressing HSPA6 (*p* < 0.001), whereas curcumin reduces the expression of HSPA6 (*p* < 0.01). Subsequently, CCK‐8 results reveal that the overexpression of HSPA6 decreased CoCl_2_‐induced HUVECs viability (*p* < 0.01), whereas treatment with curcumin increased cell viability (*p* < 0.001), which was reduced by the overexpression of HSPA6 (*p* < 0.001; Figure 4C). Meanwhile, in CoCl_2_‐treated HUVECs, overexpression of HSPA6 leads to increased expression of MMP1 and MMP13 (*p* < 0.001) and decreased expression of TIMP‐1 (*p* < 0.01); however, curcumin treatment reversed this effect (*p* < 0.01; Figure 4D). In addition, overexpression of HSPA6 induced increased apoptosis in CoCl_2_‐treated HUVECs (*p* < 0.01), whereas curcumin treatment reduced the apoptosis rate induced by HSPA6 (*p* < 0.01; Figure 4E). Cell cycle assays reveal that the overexpression of HSPA6 induced cell cycle arrest at the G2 phase (*p* < 0.001), which was reversed by treatment with curcumin (*p* < 0.001; Figure 4F). These results suggest that the regulatory effects of curcumin on viability, apoptosis, and cell cycle in CoCl_2_‐treated HUVECs depend on the expression of HSPA6.

**FIGURE 5:**
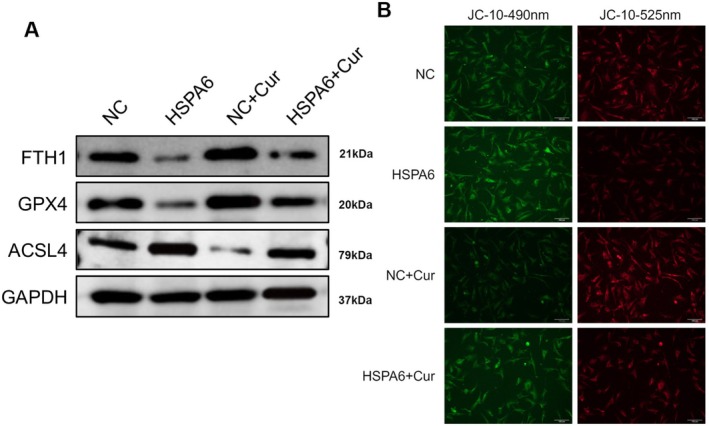
Curcumin inhibited ferroptosis and mitochondrial damage in CoCl_2_‐induced HUVECs by HSPA6. The HUVECs were transfected with HSPA6 vector and were subsequently pre‐incubated with 100 μM CoCl_2_ for 12 h, followed by 20 μM curcumin for 12 h. (A) The protein expression of FTH1, GPX4, and ACSL4 was determined by western blotting. (B) Mitochondrial membrane potential was performed by JC‐10 staining (Scale bar: 100 μm).

We also analyzed ferroptosis indicators in endothelial cells. The results showed that overexpression of HSPA6 in CoCl_2_‐treated HUVECs decreased the protein expression of FTH1 and GPX4 and increased the protein expression of ACSL4, suggesting that HSPA6 aggravated the ferroptosis process; in contrast, curcumin treatment reversed the expression of these proteins (Figure 5A). Furthermore, the results of mitochondrial membrane potential assessment are displayed in Figure 5B. Overexpression of HSPA6 increases green fluorescence intensity and decreases red fluorescence intensity in CoCl_2_‐treated HUVECs, indicating mitochondrial collapse. In contrast, curcumin treatment can increase red fluorescence intensity and decrease green fluorescence intensity. These results demonstrate that curcumin inhibits CoCl_2_‐induced ferroptosis and mitochondrial damage by regulating HSPA6 expression.

### 4.5 HSPA6 Impaired Endothelial Cells by Regulating Ferroptosis Process

**FIGURE 6:**
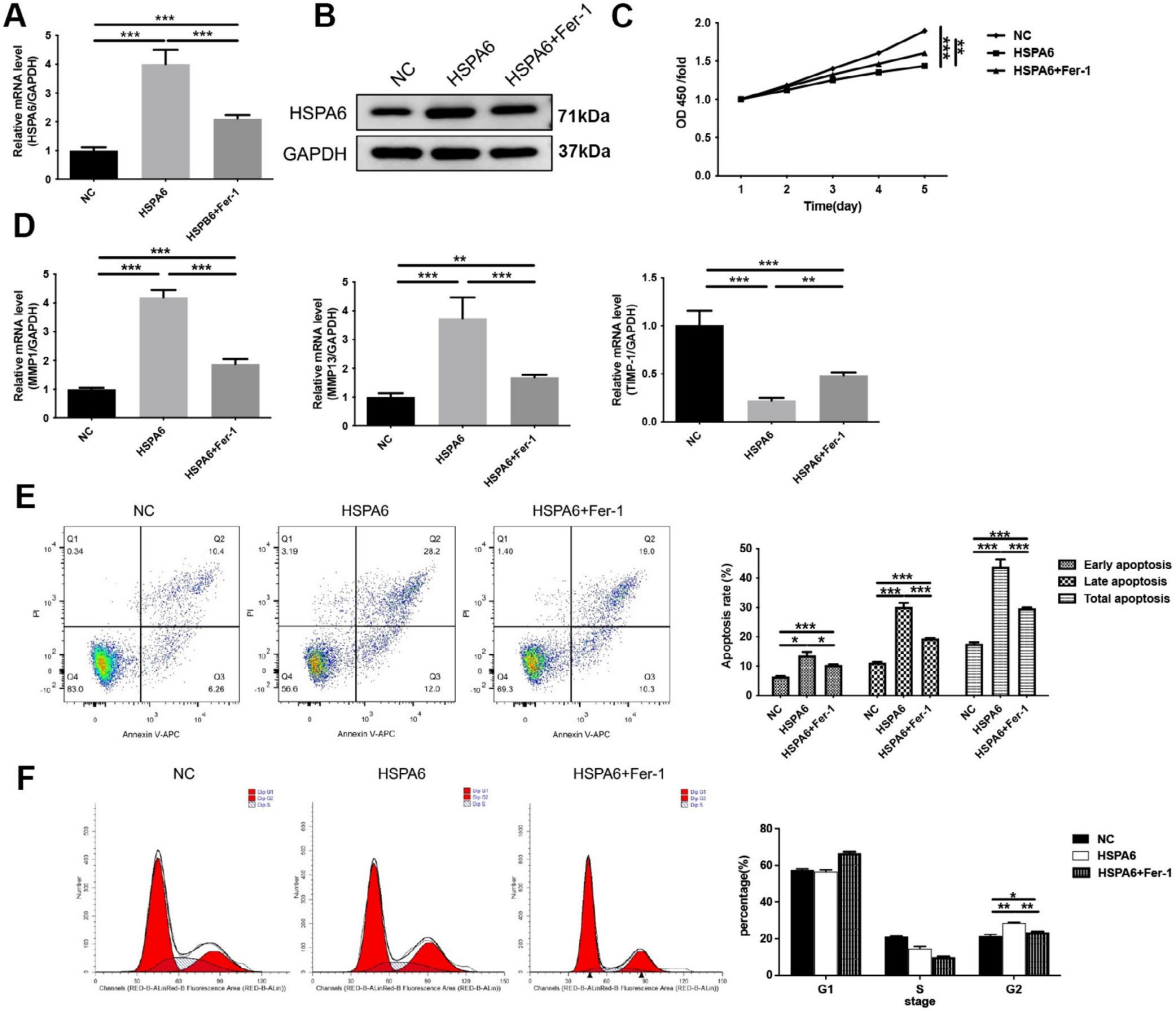
HSPA6 inhibited proliferation in CoCl_2_‐induced HUVECs by regulating the ferroptosis process. The HUVECs were transfected with HSPA6 vector and were subsequently pre‐incubated with 100 μM CoCl_2_ for 12 h, followed by 1 μM Ferrostatin‐1 (Fer‐1) for 12 h. (A) The mRNA expression of HSPA6 was measured by RT‐qPCR. (B) The protein expression of HSPA6 was determined by western blotting. (C) CCK8 was used to assess cell viability. (D) The mRNA expression of MMP1, MMP13 and TIMP‐1 was measured by RT‐qPCR. (E) Apoptosis was analyzed by flow cytometry. (F) Cycle progression was analyzed by flow cytometry. *p < 0.05, **p < 0.01, ***p < 0.001.

To elucidate the mechanism of HSPA6‐induced endothelial cell injury, we introduced an inhibitor of ferroptosis (Ferrostatin‐1, Fer‐1) to CoCl_2_‐treated HUVECs for a rescue assay. The purpose is to investigate whether HSPA6 influenced vascular endothelial cell injury through ferroptosis. RT‐qPCR and WB results suggest that overexpression of HSPA6 increases the expression of HSPA6 in HUVECs (*p* < 0.001), which is downregulated by Fer‐1 (*p* < 0.001; Figure 6A,B). In addition, Fer‐1 increased cell viability that had been downregulated by HSPA6 in CoCl_2_‐treated HUVECs (*p* < 0.01; Figure 6C). Later, the expression of matrix metalloproteinase was also analyzed. Compared to the HSPA6 group, the HSPA6 + Fer‐1 treatment significantly decreases the expression of MMP1 and MMP13 (*p* < 0.001), while increasing the expression of TIMP‐1 (*p* < 0.01; Figure 6D). These results indicate that Fer‐1 has an inhibitory effect on matrix metalloproteinase activity and extracellular matrix degradation in HUVECs. Furthermore, in CoCl_2_‐treated HUVECs cells, we found that Fer‐1 inhibited HSPA6‐induced apoptosis (*p* < 0.001) and increased cell cycle progression (*p* < 0.01; Figure 6E,F). Collectively, these results suggest that HSPA6 could downregulate cell viability, block the cell cycle, and enhance apoptosis to induce endothelial cell injury by promoting the ferroptosis process.

**FIGURE 7:**
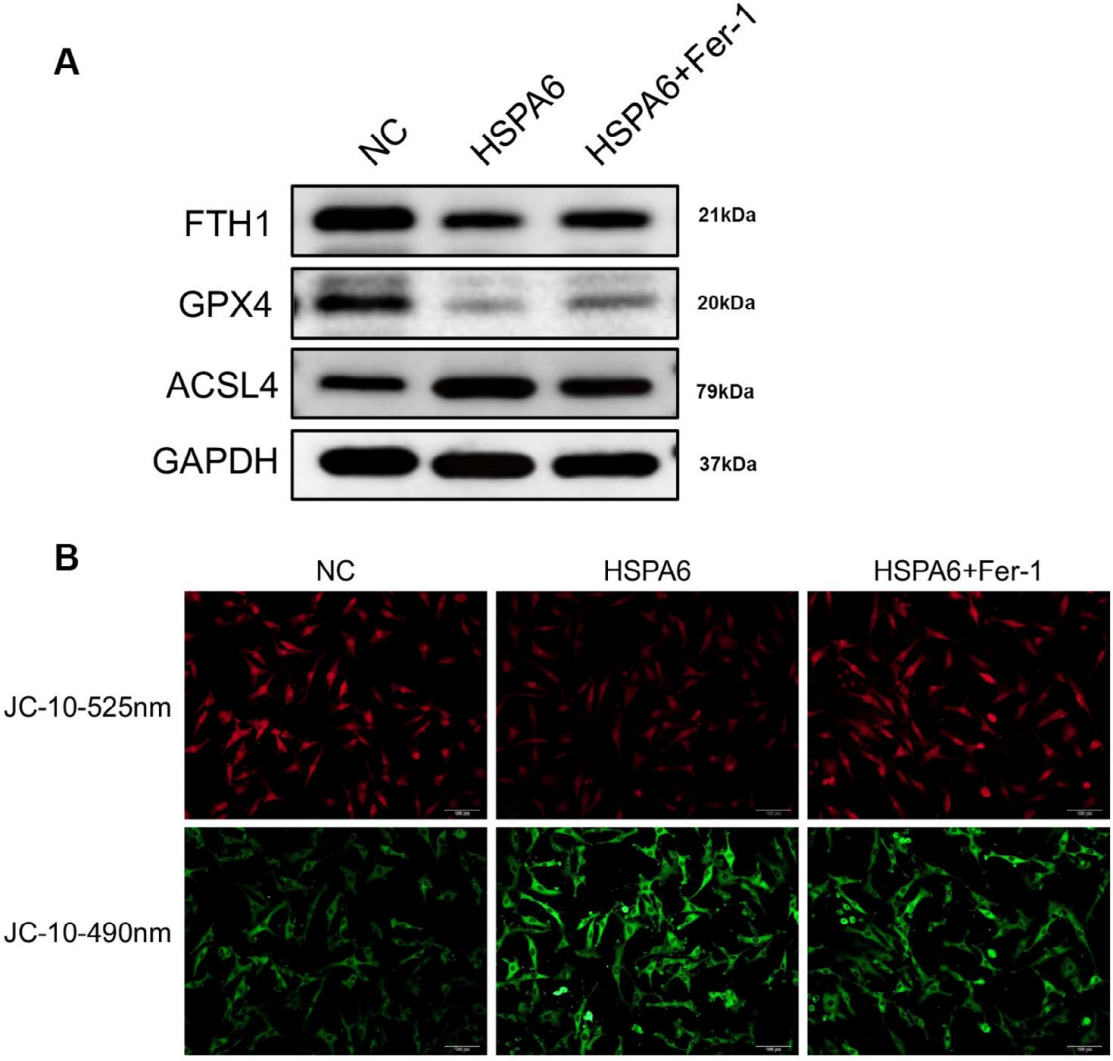
HSPA6 enhanced ferroptosis and mitochondrial damage in CoCl_2_‐induced HUVECs by regulating the ferroptosis process. The HUVECs were transfected with HSPA6 vector and were subsequently pre‐incubated with 100 μM CoCl_2_ for 12 h, followed by 1 μM Ferrostatin‐1 (Fer‐1) for 12 h. (A) The protein expression of FTH1, GPX4, and ACSL4 was determined by western blotting. (B) Mitochondrial membrane potential was performed by JC‐10 staining (Scale bar: 100 μm).

Next, markers of ferroptosis were also analyzed. In CoCl_2_‐treated HUVECs, Fer‐1 downregulates the expression of ACSL4 and increases the expression of FTH1 and GPX4 compared to the HSPA6 group (Figure 7A). In addition, we also discovered that HSPA6 exacerbated mitochondrial collapse, as evidenced by the reduction in red fluorescence and the increase in green fluorescence, while Fer‐1 treatment reversed this trend (Figure 7B). In summary, these results demonstrate that the overexpression of HSPA6 induces mitochondrial collapse and exacerbates apoptosis in CoCl_2_‐treated HUVECs by enhancing the ferroptosis process.

## 5 Discussion

The growing body of evidence underscores the prevalence of hypoxic injury as a common disorder associated with numerous pathological processes. As an integral component of the vascular endothelium, endothelial cells (ECs) are exquisitely sensitive to the deleterious effects of hypoxia, which can precipitate a spectrum of vascular diseases (Majewska et al. 2021). Curcumin, a traditional Chinese medicine monomer, has emerged as a potent protector of vascular endothelial cells against hypoxia‐induced death (Mohandas and Rangasamy 2021). To our knowledge, this study pioneers in demonstrating that curcumin effectively mitigates CoCl_2_‐induced apoptosis, ferroptosis, and mitochondrial collapse, and enhances cell viability in HUVEC by modulating the expression of HSPA6. The vascular protective effects of curcumin have also been demonstrated in animal models of hypoxia‐induced vascular endothelial injury. For instance, existing studies have shown that curcumin alleviates cerebral and vascular endothelial damage in a chronic intermittent hypoxia model (Wang et al. 2018). In thrombosis models, curcumin treatment has been found to promote the resolution of deep vein thrombi in mice (T. Wang et al. 2021). These findings indirectly support the biological relevance of our in vitro observations.

To elucidate the protective effects of curcumin on vascular endothelial cells, we recapitulated a hypoxic milieu by treating HUVECs with CoCl_2_, a chemical mimetic of hypoxia that engenders a hypoxic state under normoxic conditions by inhibiting intracellular prolyl hydroxylase (Chen et al. 2018). Our findings mirrored previous reports, revealing that CoCl_2_ triggers apoptosis, arrests the cell cycle at the G2 phase, and impedes cell proliferation in HUVECs (Wu et al. 2022; Yang et al. 2016). Notably, curcumin treatment counteracted these effects by promoting cell proliferation, cycle progression, and suppressing apoptosis, thereby underscoring its cytoprotective role. These observations are consonant with prior studies documenting curcumin's ability to bolster cell proliferation, thwart apoptosis, and safeguard endothelial cells from damage (Li et al. 2016; Yuan et al. 2022). Matrix metalloproteinases (MMP1 and MMP13) are involved in extracellular matrix degradation and tissue remodeling. TIMP‐1 is a matrix metalloproteinase inhibitor that binds to and inhibits the activity of matrix metalloproteinases. Under hypoxia, HUVEC cells modulate the composition and structure of the extracellular matrix by activating related signaling pathways or transcription factors, resulting in the up‐regulation of MMP1 and MMP13 expression and the downregulation of TIMP‐1 expression (Lee et al. 2012; Luo et al. 2024). In the current study, it indicates that curcumin reverses this trend under hypoxic conditions, downregulates MMP1 and MMP13 while upregulates TIMP‐1, thereby suggesting its involvement in regulating matrix metalloproteinase activity and extracellular matrix degradation.

Beyond apoptosis, ferroptosis has garnered increasing attention as a cell death mechanism relevant to vascular endothelial injury. Hypoxia has been shown to stimulate ferroptosis in vascular endothelial cells, lead to endothelial damage (Lei et al. 2022). In line with these findings, our study demonstrates that hypoxic HUVECs exhibit heightened intracellular iron content and enhanced expression of ferroptosis markers, which is reversed by curcumin treatment, thereby blocking ferroptosis. This aligns with previous studies underscoring curcumin's resistance to ferroptosis (Yang et al. 2021; Zhou et al. 2023). In addition, mitochondria play a crucial role in cellular homeostasis. The ferroptosis is associated with mitochondrial dysfunction, conversely, mitochondrial dysfunction induces the onset of ferroptosis (Li, Jia, et al. 2023). Accordingly, the present study was conducted to explore the mitochondrial function. The results reveal that HUVECs under hypoxic conditions has lower mitochondrial membrane potential, suggesting mitochondrial collapse. And curcumin treatment improves mitochondrial function and alleviates mitochondrial damage in HUVECs. This also suggests that curcumin improves cellular homeostasis by restoring mitochondrial function.

To identify the key genes underpinning curcumin's efficacy, we performed transcriptome sequencing and RT‐qPCR analysis. Notably, HSPA6 expression is significantly upregulated by CoCl_2_ and downregulated by curcumin. Consequently, HSPA6 was selected for further investigation as a curcumin‐regulated downstream gene. Belonging to the heat shock protein family, HSPA6 is expressed in microvascular endothelial cells and smooth muscle cells (Liu et al. 2008). Our study reveals that overexpression of HSPA6 exacerbates cellular injury in CoCl_2_‐induced HUVECs by inhibiting cell proliferation and cell cycle progression, and induced ferroptosis, apoptosis, and mitochondrial damage. These findings are congruent with previous studies reporting HSPA6's inhibitory effects on cell proliferation and DNA replication (Fu et al. 2022). Additionally, our study demonstrated for the first time that curcumin exerts its cytoprotective function by regulating the expression of HSPA6, which could be utilized in pharmacological studies as a newly identified target of curcumin.

In addition to HSPA6, our transcriptomic analysis identified several other differentially expressed genes, including COL1A1, COL3A1, THBS2, VCAN, and POSTN. Among these, COL1A1 and COL3A1 are established markers of interstitial remodeling. Previous studies have reported significant upregulation of COL1A1 and COL3A1 in pulmonary microvascular endothelial cells under hypoxic conditions, contributing to extracellular matrix (ECM) remodeling in pulmonary vasculature (Zhang et al. 2018). THBS2 has been shown to regulate ECM reorganization during physiological angiogenesis (Krady et al. 2008). In a model of angiotensin II‐induced aortic dilation, versican (VCAN) was among the most significantly upregulated ECM proteins, promoting aortic expansion (Fava et al. 2018). Furthermore, knockdown of POSTN was found to suppress atherosclerotic plaque formation by modulating inflammation and ECM remodeling (Schwanekamp et al. 2016). These findings collectively suggest that COL1A1, COL3A1, THBS2, VCAN, and POSTN are involved in ECM‐related processes. Notably, vascular injury response is closely linked to ECM remodeling. Endothelial injury can trigger further ECM expansion, leading to a granulation tissue‐like response that initiates angiogenesis and provisional ECM deposition. Such altered matrix composition, in combination with the availability of growth factors and mediators, activates fibroblasts, inflammatory cells, and Sca1 progenitor cells, thereby stimulating a pro‐fibrotic ECM response characterized by substantial deposition of interstitial collagens. This cascade ultimately exacerbates vascular endothelial injury and contributes to the pathogenesis of cardiovascular diseases (Lin and Davis 2023). Therefore, these genes are also likely to play roles in regulating endothelial cell injury under hypoxic conditions, though their specific mechanisms of action require further investigation in future studies.

However, the CoCl_2_‐induced vascular endothelial hypoxia model in vitro presents differences between chemical and physiological hypoxia. Although cobalt chloride primarily mimics hypoxia by stabilizing hypoxia‐inducible factors (HIFs), it may not fully recapitulate the multifaceted pathophysiology of ischemia in vivo, such as nutrient deprivation, metabolic waste accumulation, cell–cell interactions, and hemodynamic alterations. Furthermore, this in vitro model lacks extracellular matrix components, pericytes, smooth muscle cells, or immune cells—all of which play critical roles in angiogenesis, vascular remodeling, and thrombus formation in vivo. It is also important to note that the concentration and duration of CoCl_2_ exposure require precise control, as excessively high concentrations or prolonged treatment may induce nonspecific cytotoxicity that is not representative of physiological or pathological hypoxia. Nevertheless, the CoCl_2_ model was selected for this study due to its advantages in controllability, reproducibility, and suitability for mechanistic exploration, allowing preliminary validation of the effects and mechanisms of curcumin. Future studies should validate the roles of curcumin and HSPA6 in more complex in vivo models, such as hindlimb ischemia or arterial thrombosis, which would also facilitate the translation of our findings.

While this study sheds light on curcumin's mechanism in alleviating vascular endothelial injury, limitations remain. Notably, animal experiments are warranted to substantiate curcumin's protective effects on the vascular endothelium. On the other hand, the expression of HSPA6, a target of curcumin, in clinical samples needs to be verified to examine whether dysregulation of HSPA6 expression is associated with hypoxia‐induced vascular endothelial injury. Despite the potent anti‐ferroptotic activity of curcumin, its clinical application is limited by poor water solubility, low bioavailability, rapid metabolism, and high systemic clearance (Feng et al. 2017). Future research should focus on developing novel formulations of curcumin, such as lipid‐based nanoparticles, micelles, or nanogel‐based delivery systems, to optimize its potential as an effective clinical strategy for vascular‐related diseases.

In summary, our study underscores curcumin's vascular cell‐modulating effects and mechanisms. By targeting HSPA6 expression, curcumin promotes cell proliferation and cell cycle progression while inhibiting apoptosis, ferroptosis, and mitochondrial damage, thereby exerting a protective effect on hypoxia‐induced HUVECs. These findings provide a scientific rationale for curcumin's potential clinical application in treating hypoxia‐induced vascular diseases, such as cardiovascular diseases and venous thrombosis.

## Author Contributions

R.Y. and X.Y. contributed to the study's conception and design. R.Y., H.L., and Z.R. contributed to data collection. X.L. and X.X. contributed to statistical analysis and data visualization. R.Y. wrote the draft of the manuscript. All authors commented on previous versions. X.Y. finished the critical revision and approved the final manuscript.

## Funding

This work was supported by the Zhejiang Provincial Administration of Chinese Medicine (2022ZB146).

## Ethics Statement

The authors have nothing to report.

## Consent

The authors have nothing to report.

## Conflicts of Interest

The authors declare no conflicts of interest.

## Supporting information


Data S1.

## Data Availability

The datasets used and/or analyzed during the current study are available from the corresponding author on reasonable request.
