# Supplementary material for: Curcumin Protected CoCl2 ‐Induced Apoptosis and Ferroptosis in Human Umbilical Vein Endothelial Cells by Regulating the Expression of HSPA6
Source: Food Sci Nutr. 2026 Jan 5;14(1):e71343. doi: 10.1002/fsn3.71343 (PMC12771653; doi:10.1002/fsn3.71343)

**supplementary file: full uncropped Gels and Blots image(s)**

Figure 2A

FTH1 (21KDa): From left to right: NC->Curcumin->NC+Cocl_2_->Curcumin+Cocl_2_ -> marker


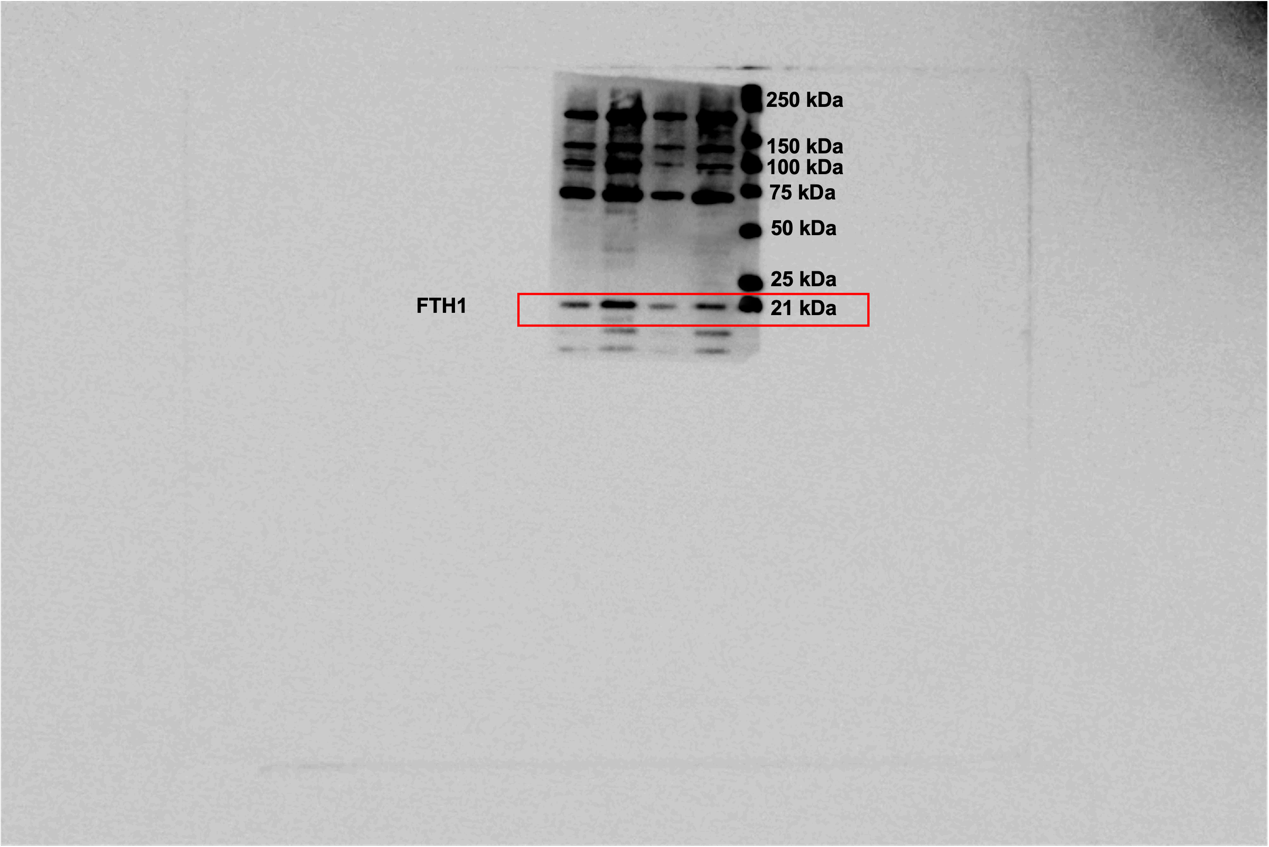


GPX4(20KDa): From left to right: marker -> NC->Curcumin->NC+Cocl_2_->Curcumin+Cocl_2_ -> marker


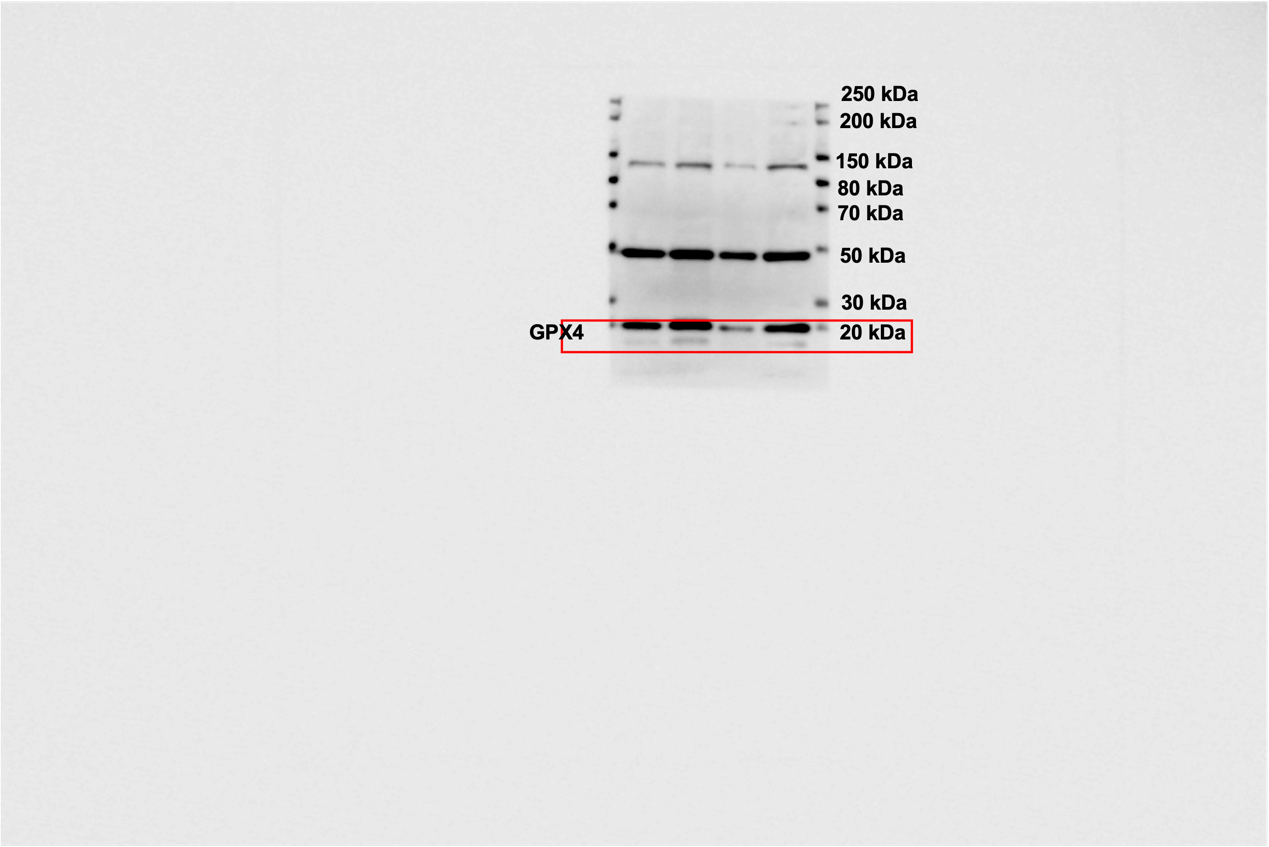


ACSL4(79KDa): From left to right: marker -> NC->Curcumin->NC+Cocl_2_->Curcumin+Cocl_2_ -> marker


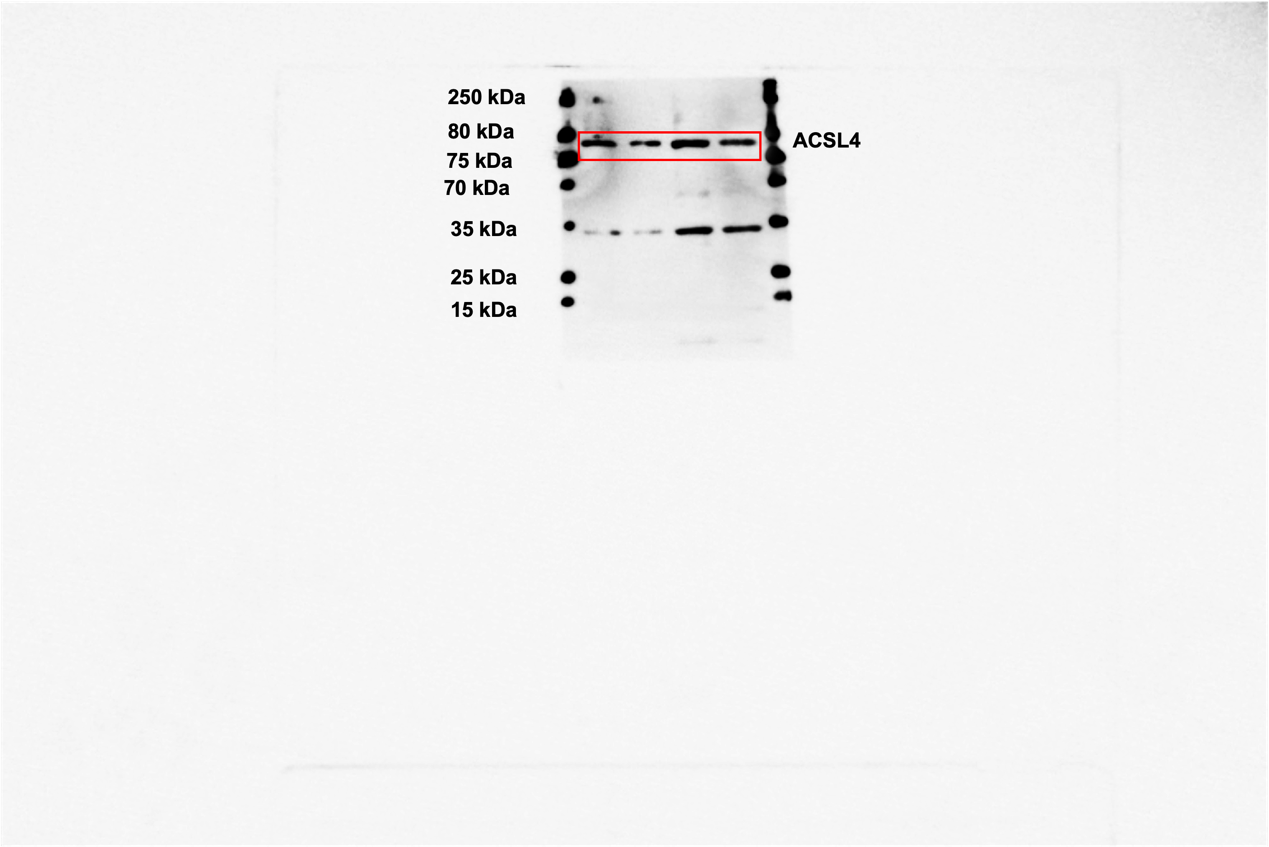


GAPDH(37KDa): From left to right: marker -> NC->Curcumin->NC+Cocl_2_->Curcumin+Cocl_2_ -> marker


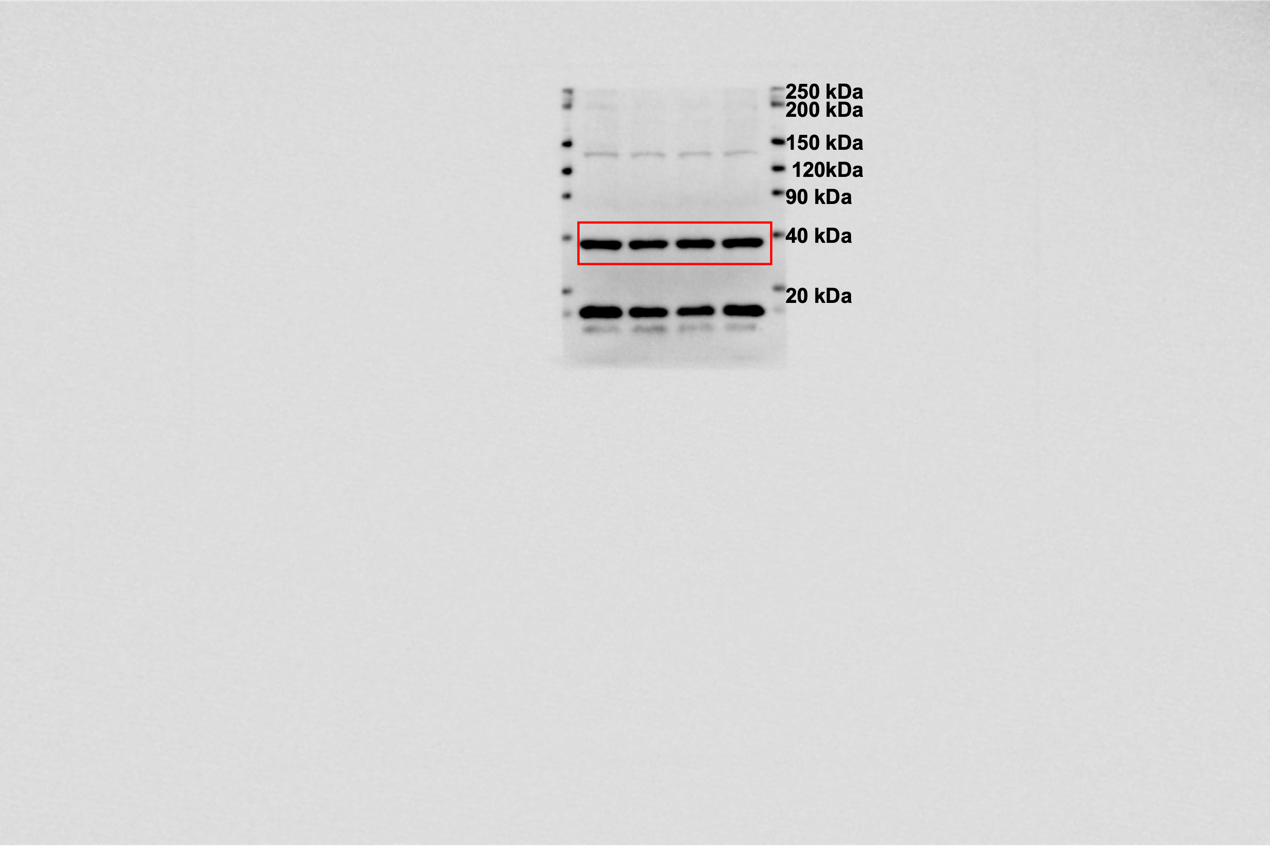


Figure 4B

HSPA6(71kDa)：From left to right: NC->HSPA6->NC+Cur-> HSPA6+Cur -> marker


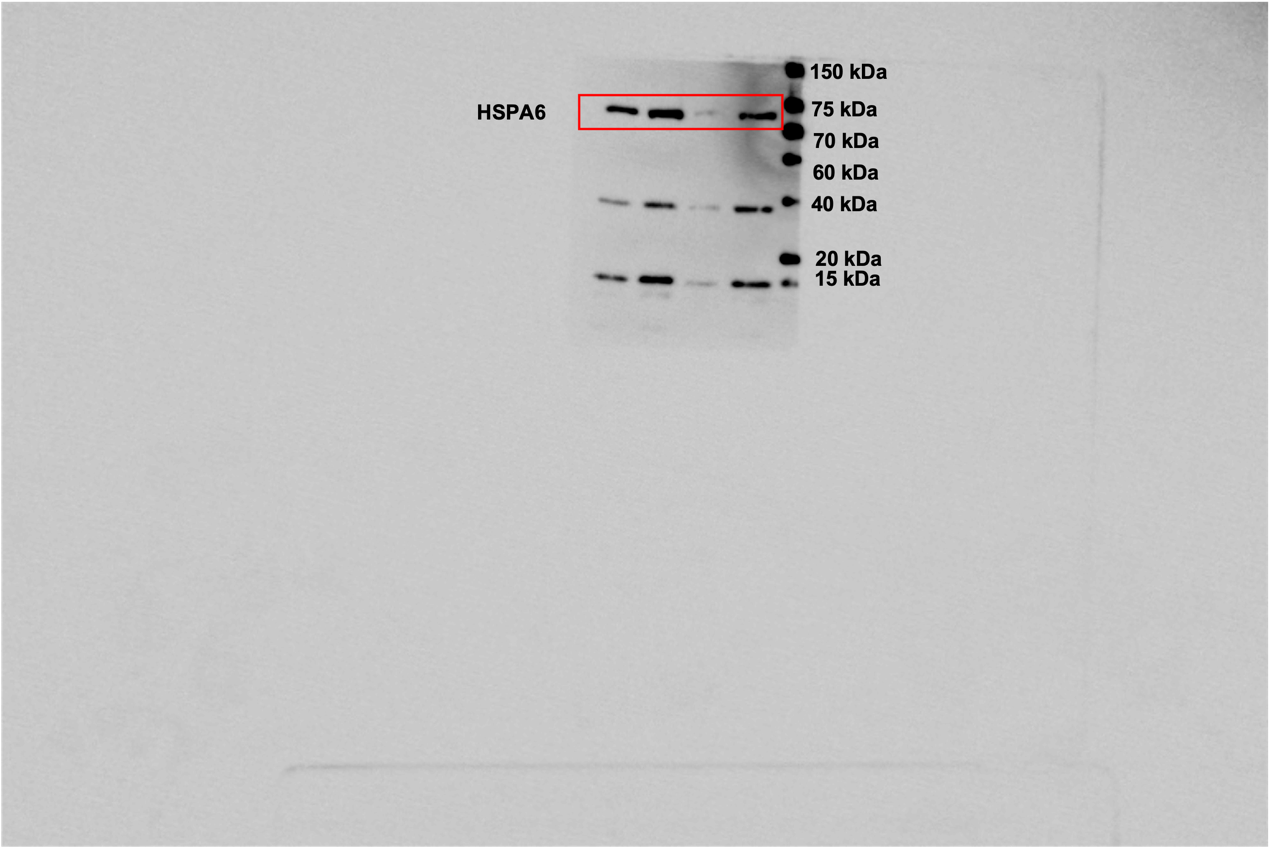


GAPDH(37kDa)：From left to right: marker -> NC->HSPA6->NC+Cur-> HSPA6+Cur -> marker


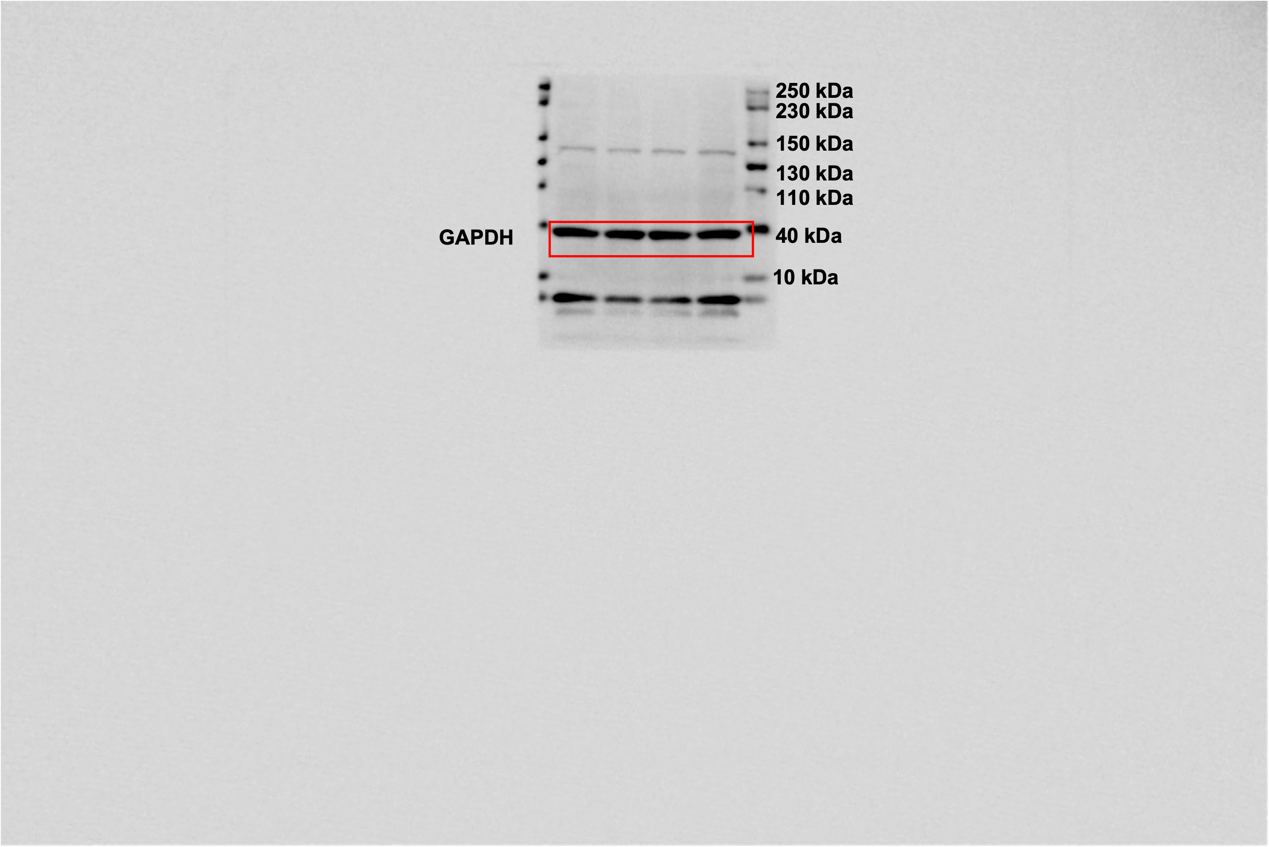


Figure 5A

FTH1(21kDa)：From left to right: marker -> NC->HSPA6->NC+Cur-> HSPA6+Cur -> marker


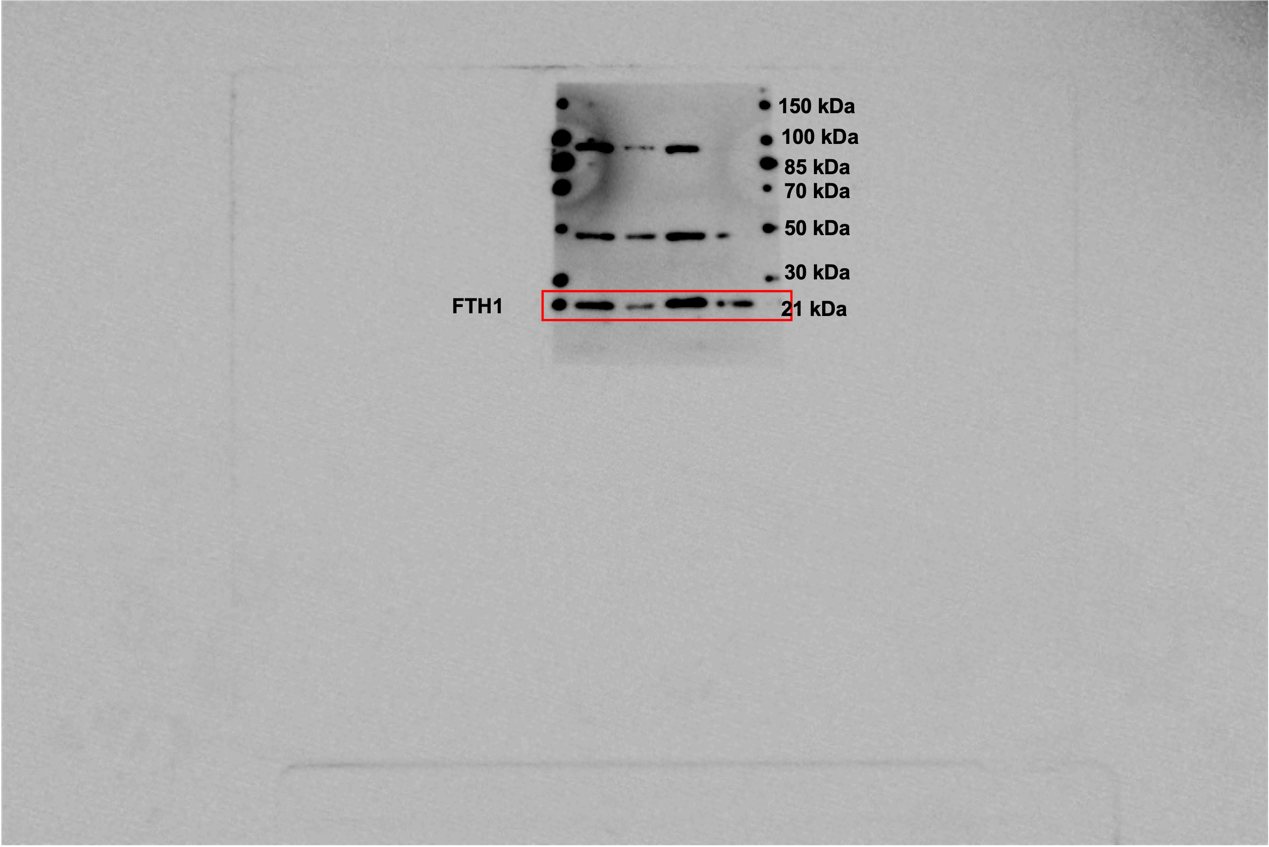


GPX4(20kDa)：From left to right: marker -> NC->HSPA6->NC+Cur-> HSPA6+Cur -> marker


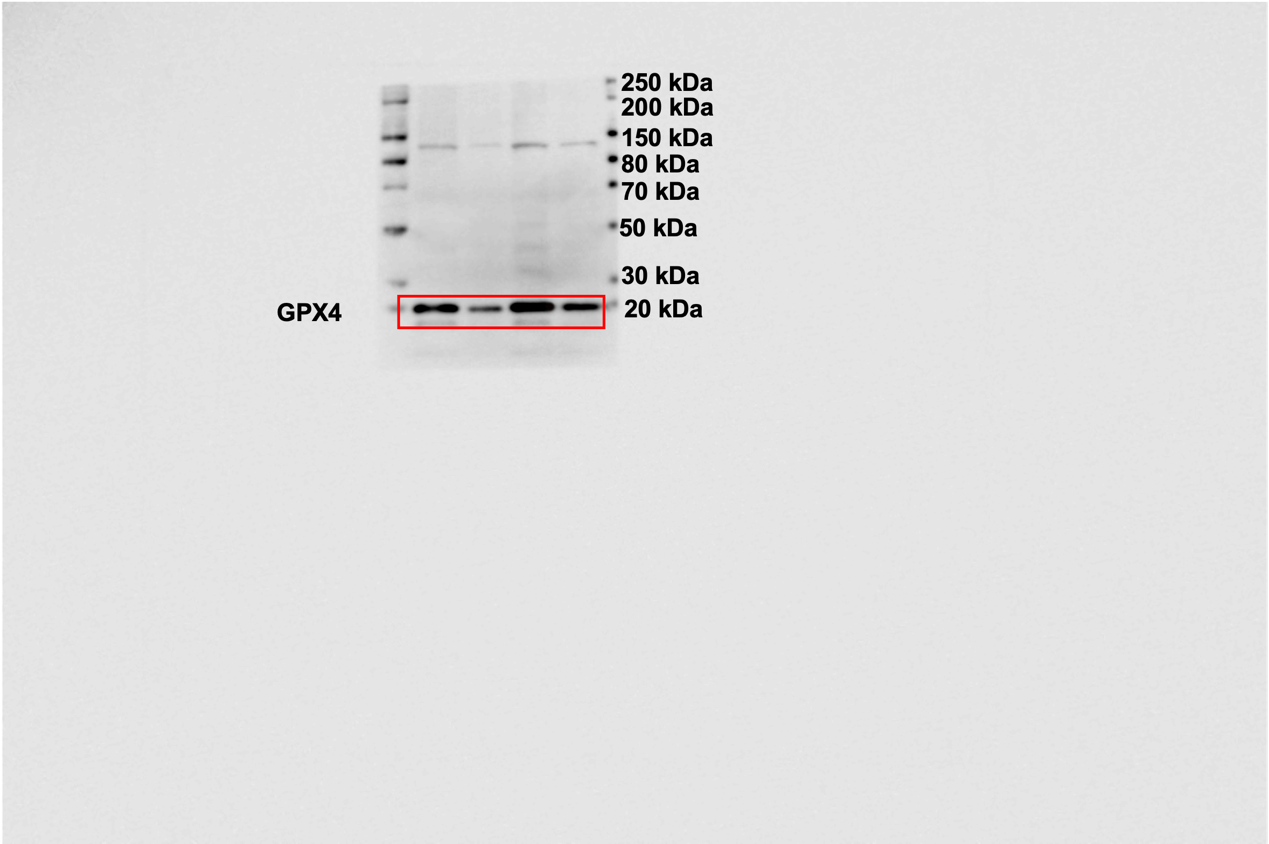


ACSL4(79kDa): From left to right: marker -> NC->HSPA6->NC+Cur-> HSPA6+Cur -> marker


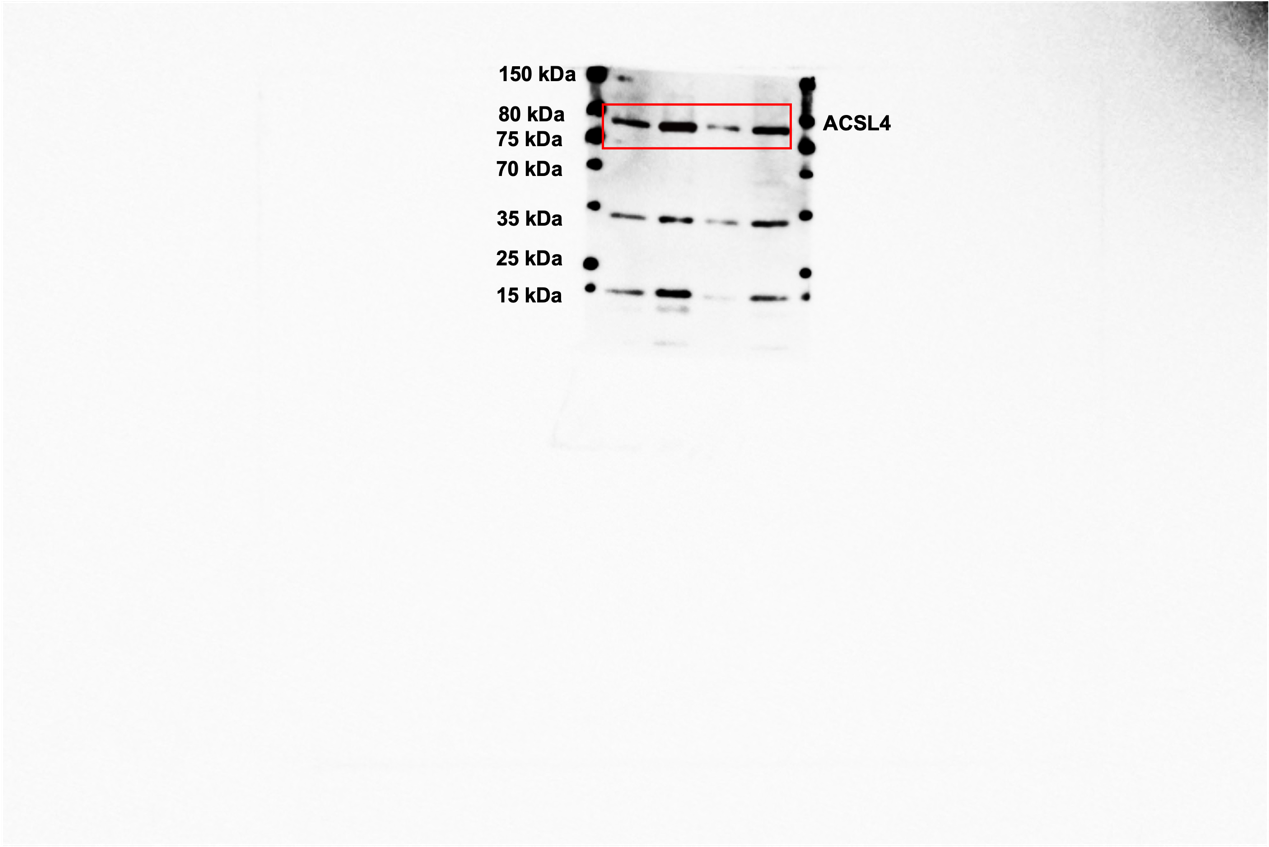


GAPDH(37kDa)：From left to right: marker -> NC->HSPA6->NC+Cur-> HSPA6+Cur -> marker


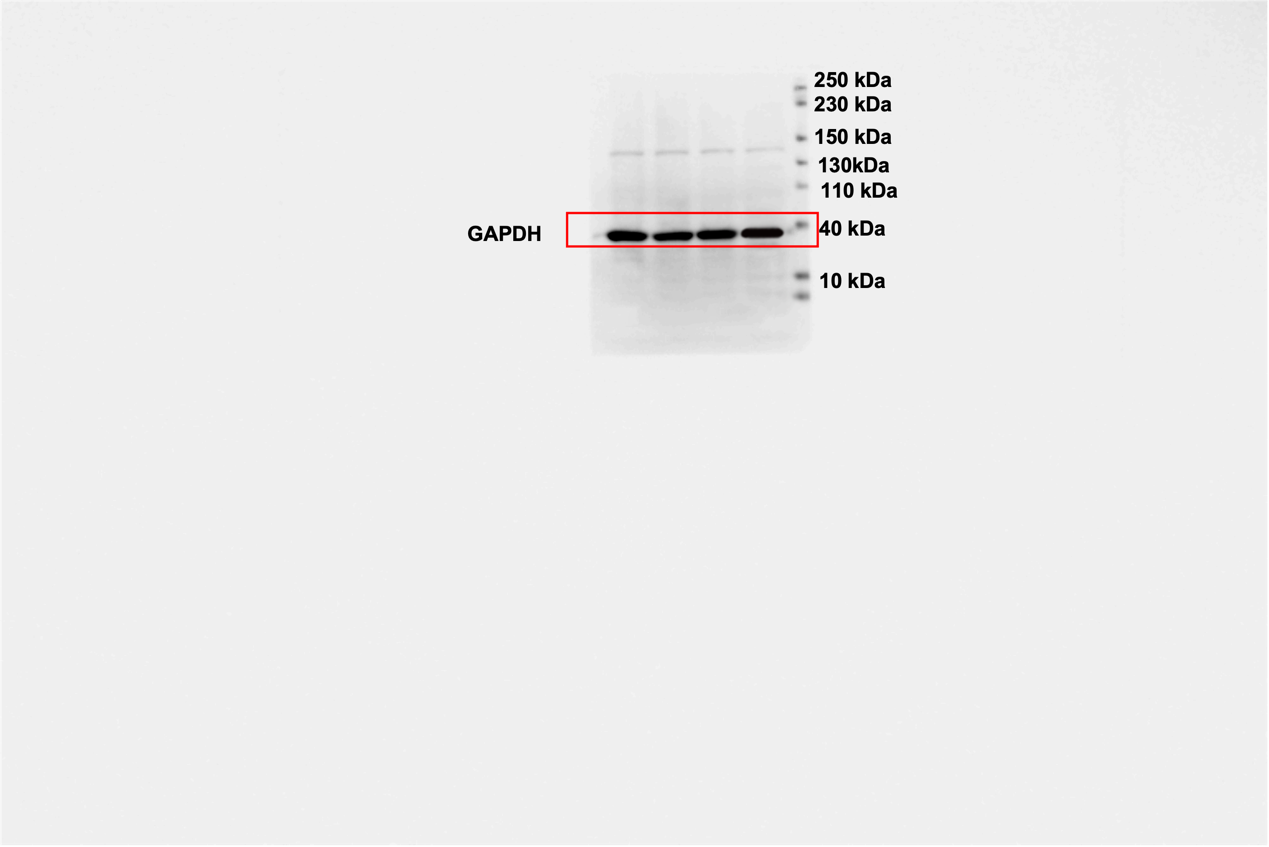


Figure 6B

HSPA6(71kDa)：From left to right: NC->HSPA6->HSPA6+Fer-1 -> marker


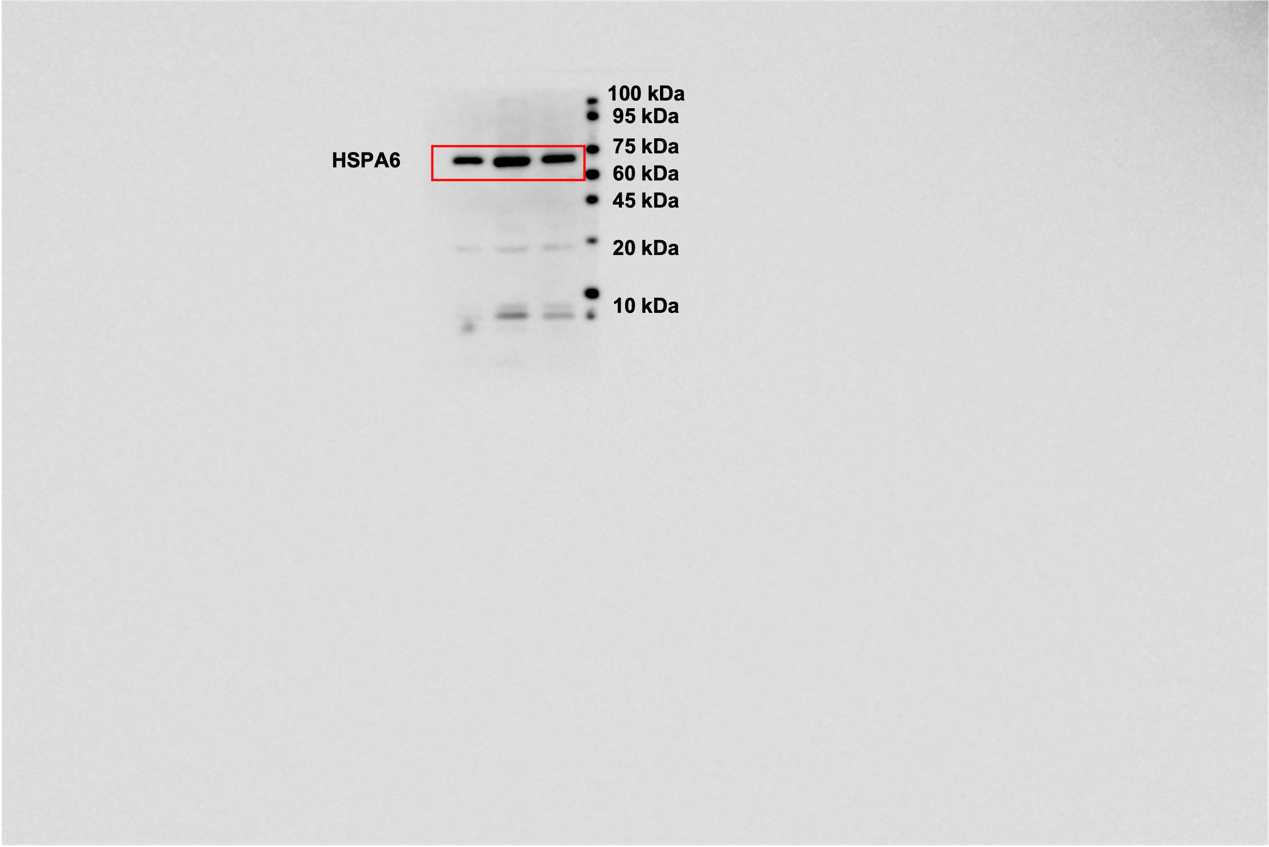


GAPDH(37kDa): From left to right: marker -> NC->HSPA6->HSPA6+Fer-1 -> marker


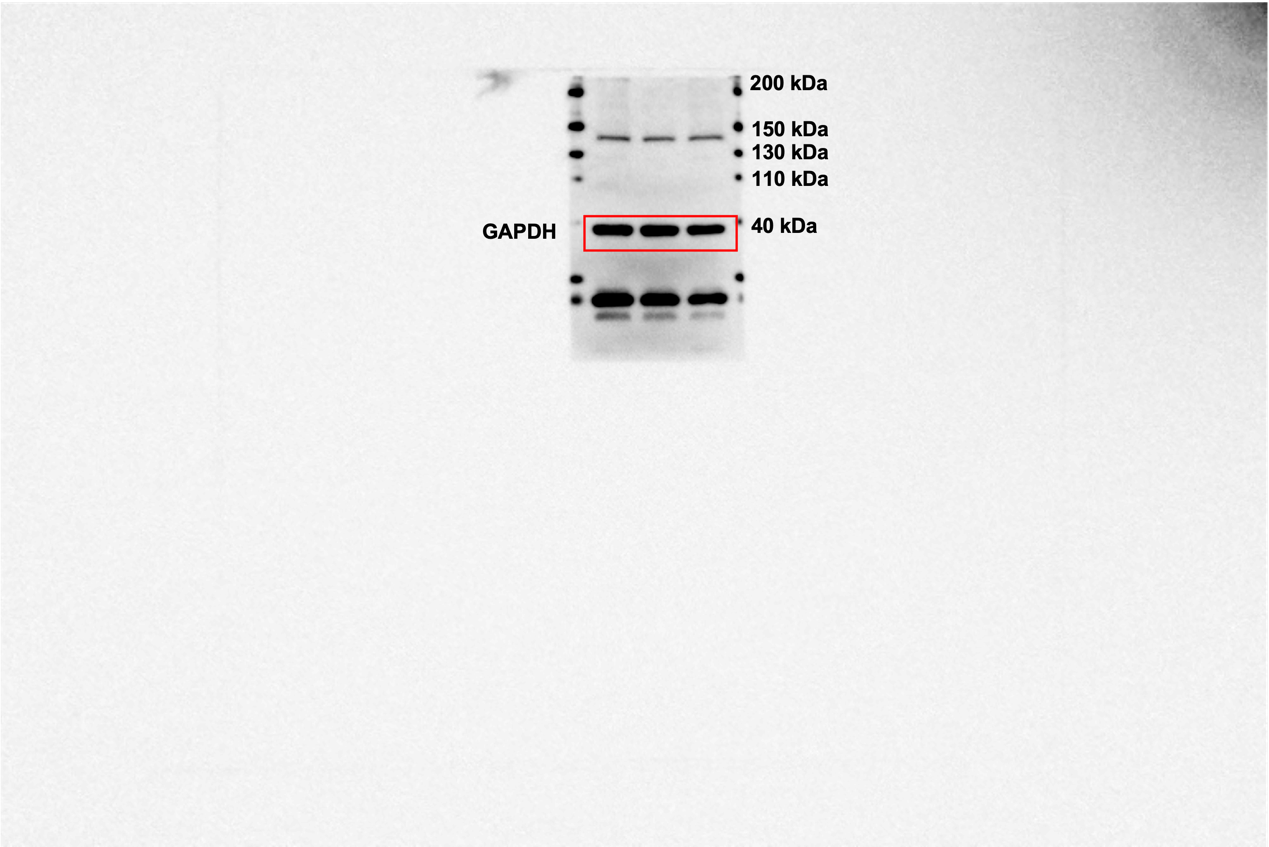


Figure 7A

FTH1(21kDa): From left to right: marker -> NC->HSPA6->HSPA6+Fer-1 -> marker


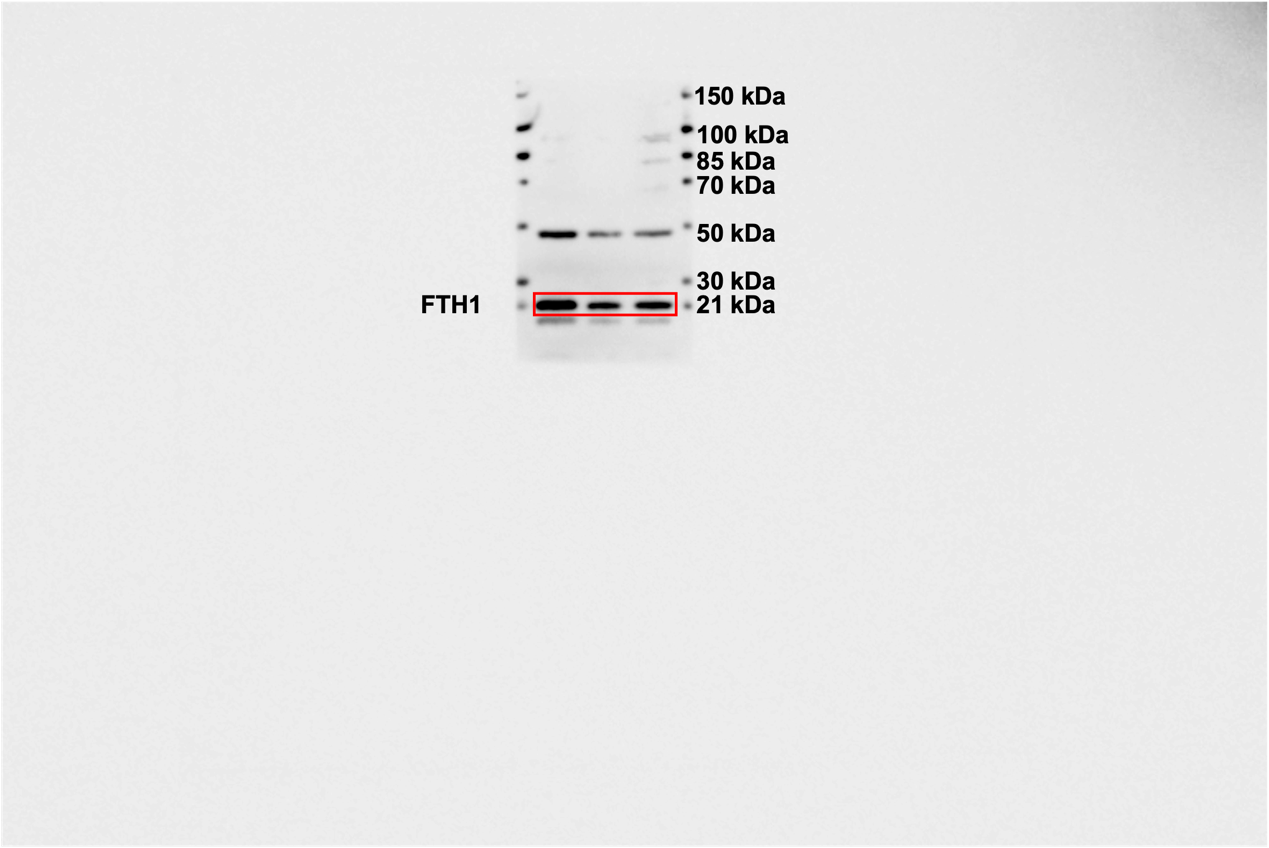


GPX4(20kDa): From left to right: marker -> NC->HSPA6->HSPA6+Fer-1 -> marker


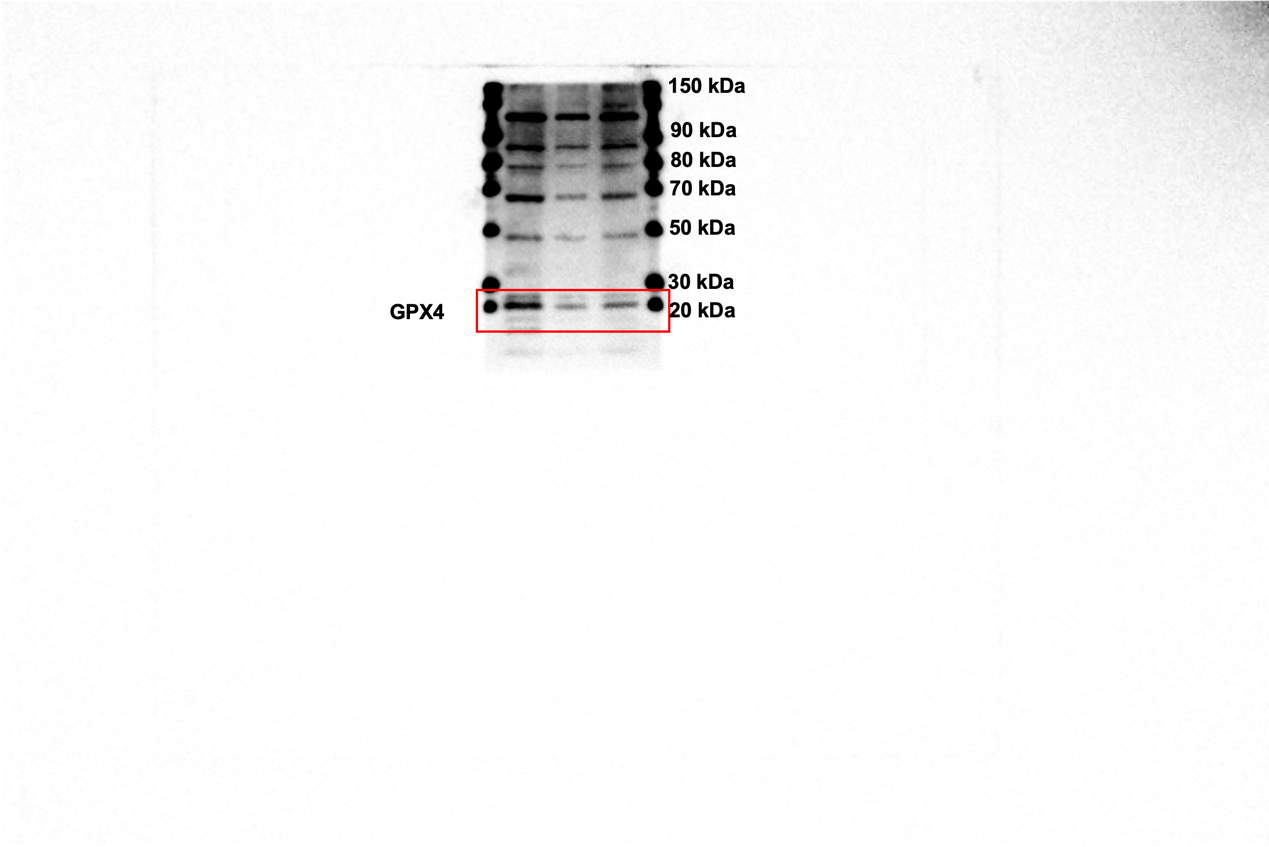


ACSL4(79kDa): From left to right: marker -> NC->HSPA6->HSPA6+Fer-1 -> marker


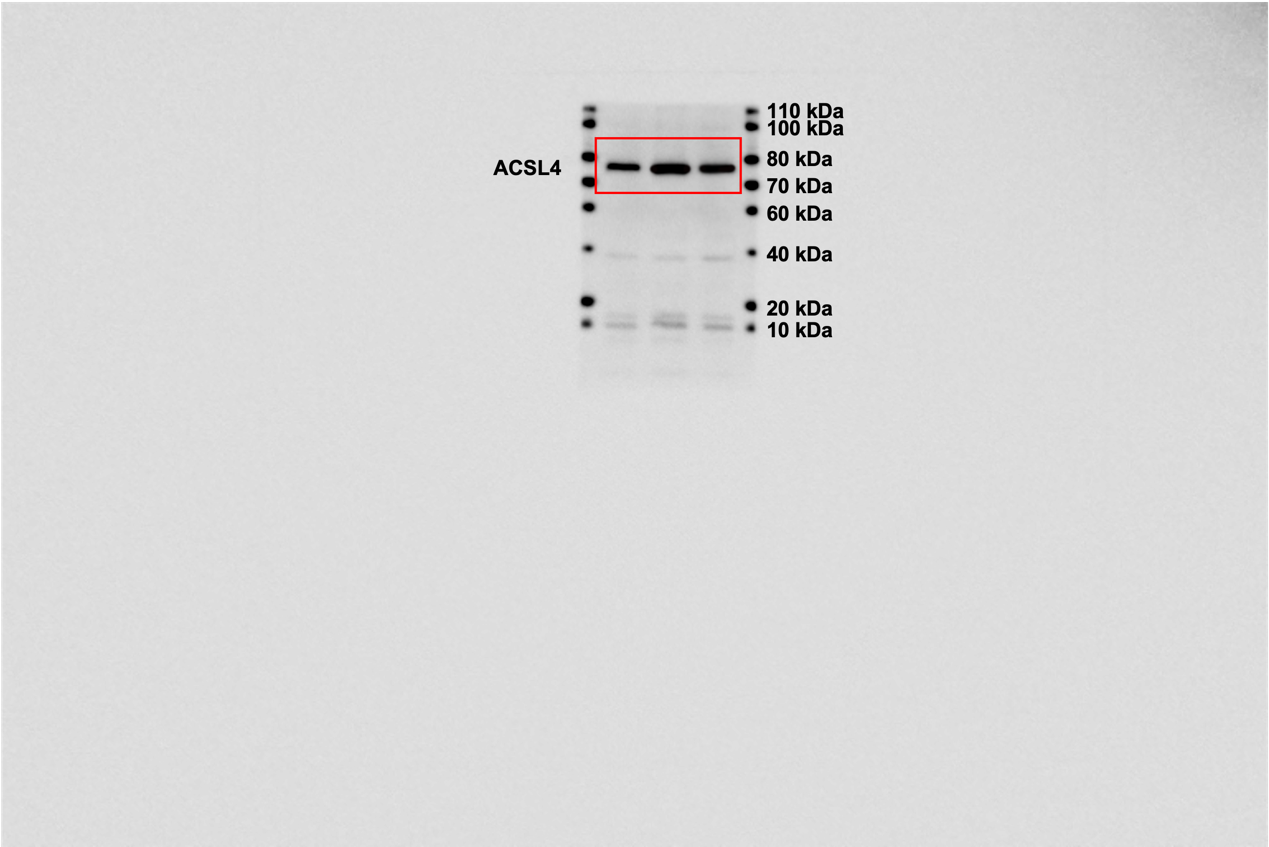


GAPDH(37kDa): From left to right: marker -> NC->HSPA6->HSPA6+Fer-1 -> marker


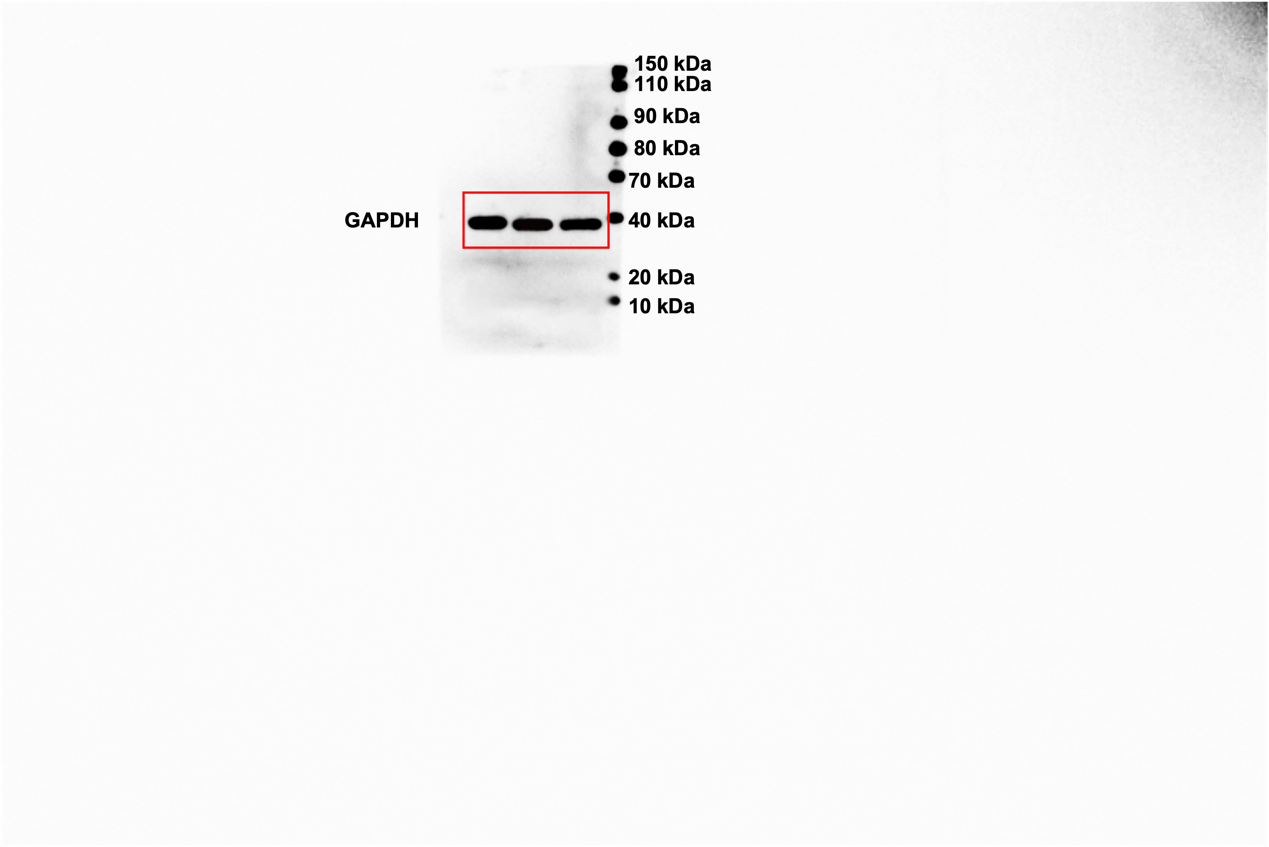

Supplement: Supplementary file 1 — Data S1. [file FSN3-14-e71343-s001.docx]
